# Transient Shifts of Incubation Temperature Reveal Immediate and Long-Term Transcriptional Response in Chicken Breast Muscle Underpinning Resilience and Phenotypic Plasticity

**DOI:** 10.1371/journal.pone.0162485

**Published:** 2016-09-09

**Authors:** Watcharapong Naraballobh, Nares Trakooljul, Eduard Murani, Ronald Brunner, Carsten Krischek, Sabine Janisch, Michael Wicke, Siriluck Ponsuksili, Klaus Wimmers

**Affiliations:** 1 Leibniz Institute for Farm Animal Biology (FBN), Institute for Genome Biology, 18196, Dummerstorf, Germany; 2 Institute of Food Quality and Food Safety, Foundation University of Veterinary Medicine, Hannover, 30173, Hannover, Germany; 3 Department of Animal Sciences, Quality of Food of Animal Origin, Georg-August-University Goettingen, 37037, Göttingen, Germany; Fred Hutchinson Cancer Research Center, UNITED STATES

## Abstract

Variations in egg incubation temperatures can have acute or long-term effects on gene transcription in avian species. Altered gene expression may, in turn, affect muscle traits in poultry and indirectly influence commercial production. To determine how changes in eggshell temperature affect gene expression, incubation temperatures were varied [36.8°C (low), 37.8°C (control), 38.8°C (high)] at specific time periods reflecting two stages of myogenesis [embryonic days (ED) 7–10 and 10–13]. Gene expression was compared between interventions and matching controls by microarrays in broiler breast muscle at ED10 or ED13 and post-hatch at day 35. Early (ED7-10) high incubation temperature (H10ΔC) resulted in 1370 differentially expressed genes (DEGs) in embryos. Ingenuity pathway analysis revealed temporary activation of cell maintenance, organismal development, and survival ability genes, but these effects were not maintained in adults. Late high incubation temperature (ED10-13) (H13ΔC) had slightly negative impacts on development of cellular components in embryos, but a cumulative effect was observed in adults, in which tissue development and nutrition metabolism were affected. Early low incubation temperature (L10ΔC) produced 368 DEGs, most of which were down-regulated and involved in differentiation and formation of muscle cells. In adults, this treatment down-regulated pathways of transcriptional processes, but up-regulated cell proliferation. Late low temperature incubation (L13ΔC) produced 795 DEGs in embryos, and activated organismal survival and post-transcriptional regulation pathways. In adults this treatment activated cellular and organ development, nutrition and small molecule activity, and survival rate, but deactivated size of body and muscle cells. Thermal interventions during incubation initiate immediate and delayed transcriptional responses that are specific for timing and direction of treatment. Interestingly, the transcriptional response to transiently decreased incubation temperature, which did not affect the phenotypes, prompts compensatory effects reflecting resilience. In contrast, higher incubation temperature triggers gene expression and has long-term effects on the phenotype. These mechanisms of considerable phenotypic plasticity contribute to the biodiversity and broaden the basis for managing poultry populations.

## Introduction

In homeotherms like birds, pre- and post-hatch development occurs only within a limited range of body temperature [1]. *In-ovo*, temperature is a critical factor affecting embryo development. Changes in eggshell temperature or inadequate timing of the proper temperature can result in morphological changes, through altered gene expression, that is fatal or produces long-term alterations in development. Under natural conditions and from the evolutionary point of view, one could argue that due to the likelihood of variation of brooding temperature under natural conditions mechanisms should have evolved to promote resilience against these unpredictable environmental factors. Indeed, in birds parent brooding causes more stable conditions than for many reptile species with shallow nests [2]. There are differences in nest temperatures varying from about 30°C to 40°C among avian species [3]. In unattended periods the nest temperature may drop considerably [4,5]. Therefore mechanisms to cope with transient lowered temperature likely exist. Such coping mechanisms may still exist in commercial broiler lines even after long-term artificial selection under highly controlled conditions.

In fact, under controlled artificial conditions in poultry production, improvement of productivity and resilience, for example to hot climates, are major issues; accordingly most research has focused on increased incubation temperature. For instance, a slightly higher egg incubation temperature has been associated with positive effects on breast meat yield (% of BW) of featherless broiler chicken [6] and muscle fiber development in turkey [7]. However, higher incubation temperature can also produce lower body weight [8]. Divergent outcomes may be attributable to differences in the intensity and duration of incubation temperature changes. Nonetheless, understanding how these changes affect development is crucial for identifying any long-term consequences and potential application of variation of incubation temperature in poultry breeding.

Also during in-utero development of mammalian species, including human, aberration of body temperature due to maternal fever may impact the post-natal life. For example, maternal fever significantly increases the risk of autism and developmental delay in humans [9]. In fact, embryonic and fetal development are periods of rapid growth and cell differentiation and predetermination of later life. Adverse environmental conditions during embryonic and fetal development provoke an adaptive response, which may lead to both persistently biased responsiveness to extrinsic factors and permanent consequences for the organismal phenotype [10,11]. The *in-ovo* development of the poultry is an ideal model for studying the impact of exogenous (physical) effects and analysing mechanisms of gene-environment interactions taking place during embryonic development and potentially affecting later life time development.

Avian myogenesis occurs in two phases during embryo development. First, between embryonic days (ED) 4 and 7, primary muscle fibers are formed from myoblasts. Next, from ED7-15, secondary muscle fibers develop around the scaffold of primary muscle fibers [12]. Fetal myoblasts are most abundant between ED8 and 12 [13]. Primary fibers represent a heterogeneous population that are committed to becoming fast (white), mixed fast/slow, and mainly slow (red) fibers, whereas secondary fibers belong to the fast myogenic lineage, i.e. the two developmental phases of myogenesis give rise to different myofibers [14]. Breast muscle is a valuable meat product in chicken. *Musculus pectoralis* consists of type II, white muscle fibers, with fast-contracting properties and high glycogen content for glycolytic metabolism. Changes in incubation temperature occurring during the transition from primary to secondary muscle fiber formation may induce gene expression changes in the embryo that result in altered muscle phenotypes. Elevated incubation temperature between ED7 and 10 positively influences slaughter and breast muscle weights in broiler males. In contrast, there was no effect on hind muscle weight, with represents a red muscle mainly consisting of type I fibers. Moreover, there was no negative effect on meat quality [15].

Manipulation of incubation temperature at specific periods may offer a method by which to improve the efficiency of broiler meat production by altering gene expression during myogenesis. This study sought to identify immediate and late transcriptomic responses in a white muscle (*Musculus pectoralis*) breast muscle following changes in incubation temperature. The identification of differentially expressed genes and their functional annotation to pathways and networks offers insight into the physiological mechanism that has evolved to cope with lower and higher incubation temperatures including those relevant to improve muscularity and heat tolerance.

## Materials and Methods

### Sample Collection

**Fig 1:**
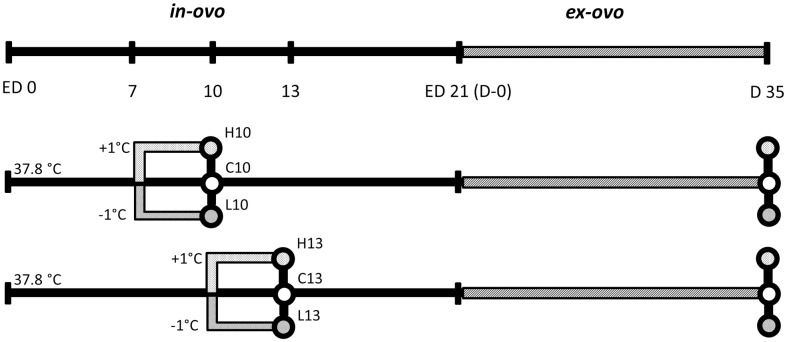
The experimental design shows the timeline and parameters for treatment. Each circle indicates the time point for sample collection (88 samples; n = 8 per treatment with adult controls n = 8 in total). H, high temperature; L, low temperature; C, control; ED, embryonic day.

Animal care procedure followed the guidelines of the German Law of Animal Protection and the experimental protocol was approved by the Institutional Animal Care and Use Committee (IACUC) of the Department of Animal Sciences of the University of Goettingen, Germany and the Leibniz Institute for Farm Animal Biology. Commercial broiler line eggs (Cobb-Vantress Inc., Siloam Springs, Arkansas, USA) were randomly selected and divided into 6 experimental groups (total number of eggs 1001) (Fig 1). Three groups had early intervention, and incubation conditions of these groups over ED7 to 10 were as follows: 1) high temperature of 38.8°C and 65% relative humidity (RH) (group H10); 2) control temperature of 37.8°C and 55% RH (C10), which equals the conditions before and after the intervention; and 3) low temperature of 36.8°C and 55% RH (L10). The remaining 3 groups had late intervention, and incubation conditions of these groups over ED10 to 13 mimicked those of the early groups, with high (H13), control (C13), and low (L13) temperatures. At ED10 and ED13 subsets of each group were obtained and breast muscles were prepared and stored for subsequent analyses. In addition to the eggs for the collection of embryo samples at ED10 or ED13, respectively, a set of eggs was treated the same way in parallel. Except for the specific treatment periods at ED7-10 or ED 10–13 these eggs were incubated at 37.8°C, 55% RH until 3 days before hatching, when RH was increased to 65% until hatch. After hatch chicks were fed a standard diet *ad libitum* until slaughter age at day 35 (D35). Broilers were slaughtered at the experimental poultry abattoir of the Department of Animal Sciences of the University of Goettingen, Germany, by electronarcosis (0.12 A, 5 to 10 sec) followed by exsanguination according to German animal welfare laws and regulations. Zoo-technical and biochemical traits were recorded. Breast tissue samples (*M*. *pectoralis)* were collected in liquid nitrogen at slaughter (D35). Embryonic samples taken at ED10 and ED13 as well as samples of D35 were sexed and for each experimental group (C10, H10, L10 and C13, H13, L13) at each time point (ED10 or ED13, respectively, plus D35) samples, balanced for sex, were selected for gene expression analyses with 8 samples per treatment (Fig 1).

### RNA Preparation

Total RNA of individual tissue samples (n = 88) was isolated by Tri-Reagent-extraction (Sigma-Aldrich, Taufkirchen, Germany). DNase treatment and column-based purification using the RNeasy Mini Kit (Qiagen, Hilden, Germany) were used to ensure purity. Quality of RNA was checked using 1% agarose gels containing ethidium bromide. Concentration of RNA was also detected by spectrometry with a NanoDrop ND-1000 spectrophotometer (PEQLAB, Erlangen, Germany). Additionally, the absence of DNA contamination was verified by using the RNA as template in standard PCR amplifying fragments of the glyceraldehyde 3-phosphate dehydrogenase (GAPDH) gene. All RNAs were stored at −80°C until further use.

### Microarray Data Processing

cDNA was generated by reverse-transcription of 500 ng of total RNA with the Ambion WT Expression Kit (Life Technologies GmbH, Darmstadt, Germany). Biotin-labeled cRNA targets were identified with Affymetrix GeneChip WT Terminal Labeling Kit (Affymetrix, Santa Clara, CA, USA). Fragmented biotin-labeled cRNAs were hybridized on Chicken Gene 1.0 ST Arrays (Affymetrix) covering 439,582 probe-sets representing 18,214 gene level probe-sets. Following staining and washing protocols, the arrays were scanned by Affymetrix GCOS 1.1.1 software for raw results with official annotation (galgal3 build 34).

### Statistical Analysis

Affymetrix Expression Console Software was used to normalize and quantify transcript expression by using the PLIER (Probe Logarithmic Intensity Error) algorithm together with DABG (detection above background), which joins probe-level p-values to create probe cell intensity values at the exon level. All data were deposited in an MIAME-compliant database, the National Center for Biotechnology Information Gene Expression Omnibus (www.ncbi.nlm.nih.gov/geo; accession number: GSE76670). Default thresholds were used to assign a “present” call (detection p-values < 0.04); present calls were synchronized with gene-level annotation. Filtering outlier and nonspecific results were done by using “genefilter” in R (www.r-project.org) considering standard deviations of normalized expression values at the gene-level. Analysis of variance was applied to detect transcriptional changes between treatment groups using the Mixed procedure in JMP Genomics (JMP Genomics 5, SAS-Institute) considering individual and combined effects of temperature, treatment period and gender and slaughter weight. Sex was excluded from the statistical model due to marginal effects. The final model included fixed effects of temperature, treatment period and interactions. Slaughter weight was included as covariate for the 35 days post-hatch time course. Transcripts with significant differences of abundance at p-values ≤ 0.05 were selected and queried for pathways analysis. At *pre-hatch* stages p < 0.05 equals FDR adjusted p-values of q < 0.15; at D35 p < 0.05 corresponding q-values ranged between 0.2 and 0.7. Moreover, we have previously shown consistency of microarray expression data with real time qPCR data using hind muscle tissue of the same animals. In fact, we obtained significant correlation coefficients ranging between 0.71 and 0.84 [16]. Annotated genes with different transcript abundances are termed “differentially expressed genes (DEGs)”; higher (lower) abundance in treated group vs. control groups is termed up-regulated (down-regulated).

### Pathway Mining

Differentially expressed genes (DEGs) with p-values and fold changes were subjected to pathway analysis using the Ingenuity Pathway Analysis software (IPA, QIAGEN Redwood City, USA). Networks, biological pathways, and gene functions of the DEGs were extracted from the IPA KnowledgeBase. Significant canonical pathways and biological functions (Fisher’s exact test, p-values < 0.05) were further adjusted for multiple testing (Benjamini-Hochberg) [17]. To simplify the interpretation of complexed biological networks, significant biological functions and pathways were aggregated into eight `major categories´ (sc. 1 –sc. 8): cell maintenance, proliferation, differentiation, and replacement (sc. 1); organism organ and tissue development (sc. 2); nutrient metabolism (sc. 3); genetic information and nucleic acid processing (sc. 4); molecular transport (sc. 5); cell signaling and interaction (sc. 6); small molecule biochemistry (sc. 7); and response to stimuli (sc. 8). Furthermore, a state of pathway regulation was indicated by “activated” with a positive Z-score or “deactivated” with a negative Z-score (IPA). Genes used to generate biological networks were selected from pathways with a significant Z-score. Legend of network shapes and relationships are available in S1 Fig.

## Results

### Effects on Breast-Muscle Transcriptome

The chicken gene 1.0 ST array contains 165,815 probe-sets representing 20,828 transcripts encoding for 18,214 genes. After data pre-processing and filtering, 8,317 entries (gene level) passed to downstream analyses. In this study, we aimed to identify the effects of embryonic incubation temperature on transcriptional changes of the muscle tissue. We hypothesized that the effects may depend on time-windows of the embryonic development more specifically the development of muscle cells. We also speculated that the effects may have an influence on muscle development post-hatch. Therefore, transcriptional profiles of the breast muscle were compared between treatment conditions and control at the embryonic (*in-ovo*) and adult (post-hatch) stages using analysis of variance. Our results showed that high temperature (38.8°C) treatment during E7-10 profoundly changed the transcriptional profile compared to the same thermal treatment during E10-13 in terms of the number of DEGs (1370 vs 365) as shown in Table 1. On the other hand, low temperature (36.8°C) treatment showed smaller effects during the same embryonic stage (E7-10) as well as the later stage of E10-13. Long-term effects of changing embryonic incubation temperature were also shown in adult stage (35 days post-hatch) with pronounced effects observed in the low thermal treatment group during E10-13 (Table 1). The further information on q-value is represented in S1 Table.

**Table 1:** Numbers of differentially expressed probes sets and respective genes (DEGs) revealed by comparisons between each *in-ovo* thermal modification condition and the time-matched control separated for embryonic stages *or* at D35 (*p*-value ≤ 0.05*).

	Treatment	Probe-sets	DEG	Regulation	
				Up	Down
**Embryo**	**H10ΔC**	1484	1370	1090	280
	**H13ΔC**	470	365	308	57
	**L10ΔC**	415	368	97	271
	**L13ΔC**	905	795	763	32
**D35**	**H10ΔC**	262	208	32	176
	**H13ΔC**	325	251	121	130
	**L10ΔC**	846	761	74	687
	**L13ΔC**	349	308	160	148

*q<0.15 at embryonic stages; q = 0.2–0.7 at D35

### Immediate and Long-Term Effects

**Fig 2:**
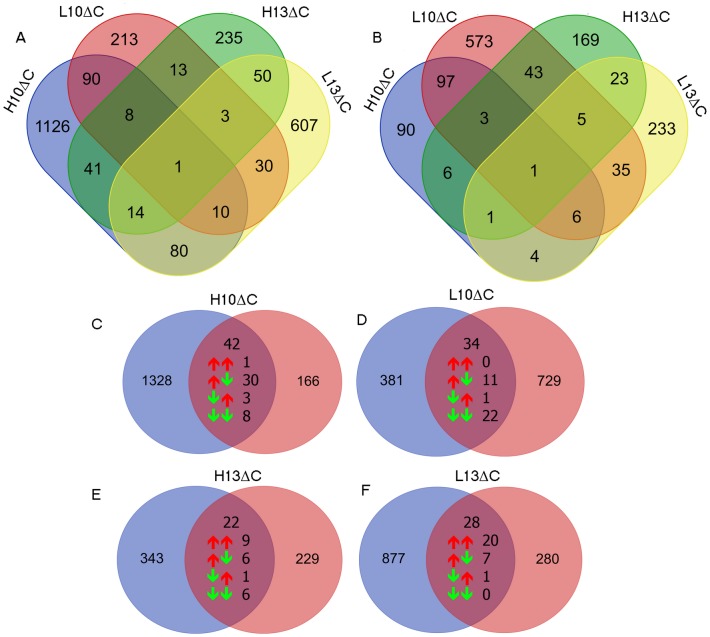
Differentially expressed genes for each treatment condition relative to controls within embryonic (A) stages and at D35 (B) and between embryonic stages and D35 within the same treatments (C-F) (blue embryonic, red adult). Intersection areas show numbers of common DEGs and their direction of regulation in each stage.

To identify immediate and long-term effects of the embryonic incubation temperature on changes of the breast-muscle transcriptional profile, we extracted common and unique DEGs from different treatment conditions as well as from the two developmental stages. Firstly, DEGs from each treatment condition compared to control were mined separately for embryonic (Fig 2A) or adult (Fig 2B) samples. Overall results revealed that the number of unique DEGs for each treatment condition is greater than that of common DEGs, suggesting that changing of the incubation temperature at a particular time-window of embryonic development affects different gene-sets and pathways. Further extraction of common DEGs between embryonic and adult samples for each treatment condition are shown in Fig 2C–2F. About 3% of the DEGs were common between the embryonic and adult samples across treatment conditions, while a majority of DEGs were unique for each stage and thermal treatment combination. The present results suggest complex biological processes and gene regulation may involve long-term effects of changing incubation temperature as well as possible environmental interactions.

To address the short- and long-term regulation of transcript expression following *in-ovo* temperature modification, DEG lists within the same treatment condition (H10, H13, L10, or L13) were compared between embryonic stages (ED10 or ED13) and D35 (Fig 2C–2F). The number of common DEGs between the two stages ranged from 22 to 42 (average 7%). Of 126 common transcripts in total (120 unique genes), 30 up and 36 down-regulated genes showed the same direction of shifting between stages (S2 Table). For groups exposed to lower temperature at either ED7-10 or ED10-13 the majority (22 out of 34 and 20 out of 28, respectively) of the DEGs found in embryonic and D35 samples showed the identical direction of change of transcript abundances compared to the matched controls; for L10ΔC most DEGs are down-regulated whereas for L13ΔC they are up-regulated. Of the 42 common DEGs of the H10ΔC at embryonic stages and D35 30 showed higher abundance in the treated samples compared to controls at embryonic stage, but lower abundance in treated than in untreated samples of D35. For H13ΔC no trends are obvious. Lowered incubation temperature leads to a higher proportion of genes that are consistently modulated over the lifetime from embryonic stages to D35; higher incubation temperature, in particular at ED7-10 results in a considerable number of genes that are diametrically shifted in immediate and late response.

### Pathway Mining

**Fig 3:**
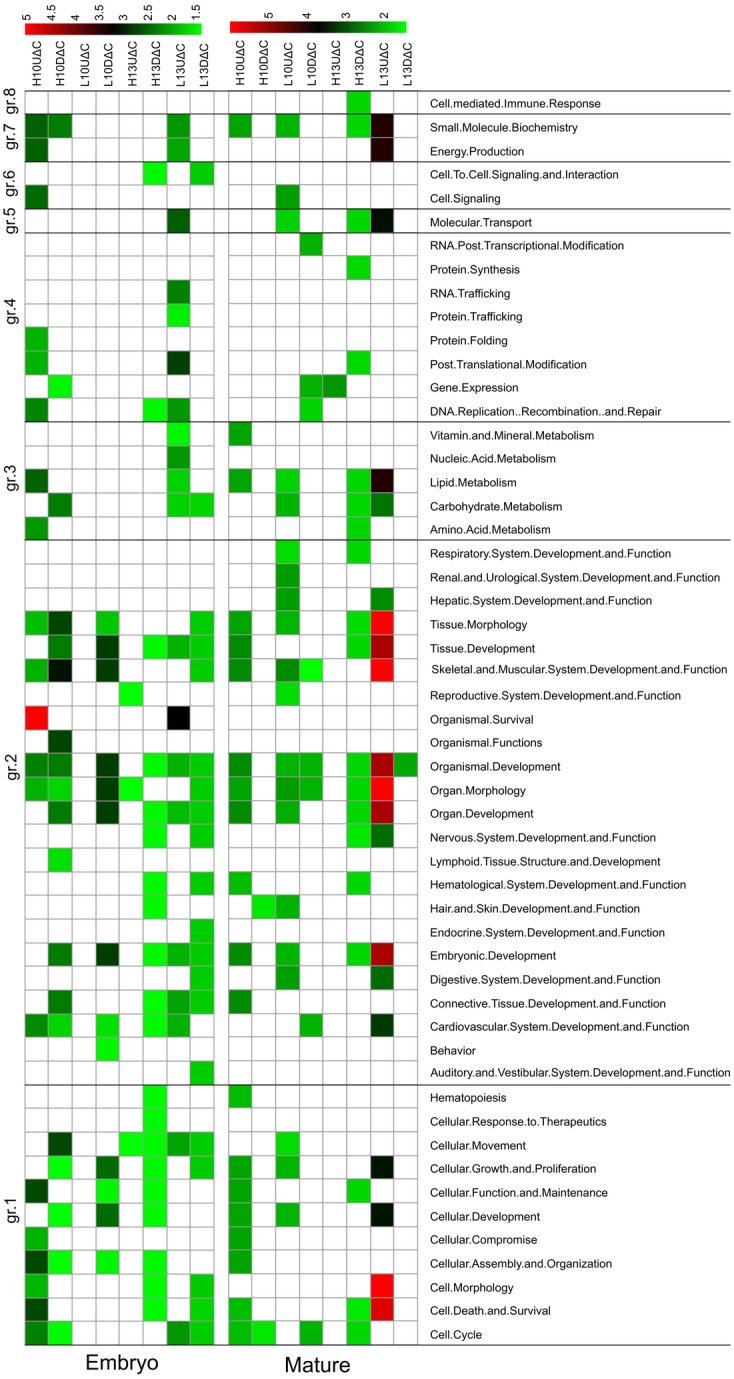
Significant pathways affected by differential gene expression at embryonic stages and D35. Up (U) or Down (D) regulation of DEG associated with each comparison (versus control) are separated within each thermal modification: increase (H) or decrease (L) in incubation temperature during ED7-10 (H10 and L10) or ED10-13 (H13 and L13). Significant pathways are grouped into eight major categories (sc.): sc.1 cell maintenance, proliferation, differentiation, and replacement; sc.2 organismal organ and tissue development; sc.3 nutrient metabolism; sc.4 genetic information and nucleic acid processing; sc.5 molecular transport; sc.6 cell signaling and interaction; sc.7 small molecule biochemistry; and sc.8 response to stimuli and associated. The—log (*p* values) related with significant pathways (Benjamini Hochberg corrected) are plotted in green (small) to red (large).

**Fig 4:**
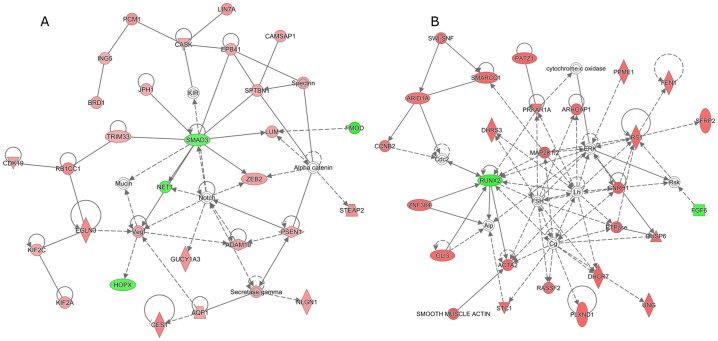
Major gene networks at ED10 and ED13. For H10ΔC a network related to tissue development and skeletal muscle development and function was derived (A). For L13ΔC a network related to skeletal-muscular and connective tissue development was derived (B). Red color, up-regulated; Green color, down-regulated.

To gain insight into molecular mechanisms underlying the thermal change effects, DEGs from each treatment (6 conditions) and stage (embryos and adults) were subjected to a knowledgebase enrichment analysis for significant biological functions and pathways using IPA. Altogether, 92 and 115 biological functions were significantly enriched for DEGs derived from embryos and adult breast-muscle samples, respectively. To summarize and simplify the results, significant bio-functions were aggregated into eight major categories based on related ontology terms. The results are shown in Fig 3 and detailed information is accessible in S3–S6 Tables. During early (ED7-10) muscle development, high embryonic incubation temperature affected biological processes including cell growth, tissue and organ development, nutrient metabolism, and cell signaling at higher degree (based on number of DEGs, bio-functions and statistically significant threshold) compared to the low temperature treatment. However, during late (ED10-13) muscle development, low temperature affected more of the aforementioned biological processes than did high temperature treatment (Fig 3). Prediction of pathway regulation as “activation” or “inactivation” based on Z-score (IPA) suggested that high incubation temperature at ED7-10 led to a shifting of cell maintenance, proliferation, differentiation, and replacement (sc. 1), and organism organ and tissue development (sc. 2) towards increased formation of cells, tissues and organs and decreased apoptosis, necrosis and death (S3 Table). Interestingly, DEGs derived from the high temperature group during early muscle development (ED7-10) revealed gene networks related to skeletal muscle development and function of which *SMAD3* (smad family member 3) functions as an down-regulated hub-gene, highly connected with other genes in the network (Fig 4A). High incubation temperature during ED10-13 tended to hamper formation of filaments, cytoskeleton and cytoplasma (S4 Table).

Long-term effects of thermal changes during embryonic development on biological processes were detected in adult samples, but less pronounced than observed in the embryos (immediate effects). Interestingly, low embryonic incubation temperature treatment seems to have long-term effects on cell growth and tissue development at later age. Low incubation temperature during ED10-13 affected most of genes related to organismal survival and post-translational modification. For L13ΔC a network related to skeletal-muscular and connective tissue development was derived (Fig 4B) with *RUNX2* (runt-related transcription factor 2) as the highly connected hub-gene. L10ΔC led to inactivation of pathways related to muscle cell formation and differentiation (S3 Table).

**Fig 5:**
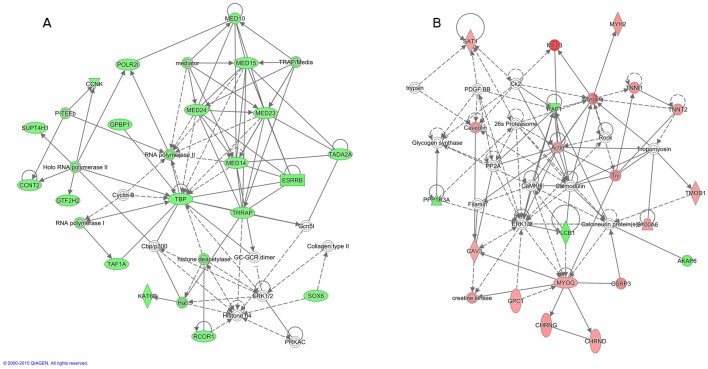
Major gene networks at D35. For L10ΔC a network related to gene expression, cell signaling and cellular function and maintenance pathways was derived (A). For L13ΔC a network related to organ, embryonic and skeletal-muscular system development and function was derived (B). Red color, up-regulated; Green color, down-regulated.

At D35, L10ΔC revealed most DEGs, however, L13ΔC affected more pathways than did early treatment (S5 and S6 Tables). Lower incubation temperature during ED10-13 (L13UΔC) significantly influenced pathways related to five major categories including cell maintenance, organismal and tissue development, nutrient metabolism, molecular transport, and small molecule biochemistry (sc.1, 2, 3, 5, and 7). Z-scores show that L13UΔC condition led to activation of most pathways that are related to anabolic functions, whereas the biofunction organismal death is strongly inactivated (S6 Table). For L10ΔC, 19 transcripts formed a network for gene expression, cellular function, and cell signaling pathways (Fig 5A). MED24 (mediator complex subunit 24), TBP (TATA box binding protein), and TRRAP (transformation/transcription domain associated protein) were identified as top candidate genes in this network. For L13ΔC, the network included organ, embryonic, and skeletal-muscular system development and function (Fig 5B). The 17 main transcripts included candidate genes of the myosin-myogenin group (MYH2, MYL3, MYOG).

The 22 durable down-regulated DEGs that are due to lower incubation temperature at ED7-10 (Fig 2D) and that are common in immediate and late response belong to gene expression pathways, and show negative Z-scores indicating deactivation. The functional network comprising these genes displays pathways of cell-to-cell signaling and interaction, cell death and survival, cell signaling, molecular transport, and vitamin and mineral metabolism (S2A Fig). For cold treatment at ED10-13 those 20 DEGs that were consistently up-regulated in short-term and long-term were related to proliferation and cell death, with Z-scores indicating deactivation of the first and activation of the second (Fig 2F). The functional network based on these DEGs is related to cell death and survival, cellular development, cellular growth and proliferation, protein synthesis, connective tissue disorders, as well as organismal injury and abnormalities (S2B Fig). For H10ΔC functional annotation analysis of 30 genes being immediately up-regulated at ED 10 but down-regulated at D35 (Fig 2C) revealed opposing regulation of biofunctions related to amino acid metabolism and hyperplasia including formation of type II myofibers.

## Discussion

The study aimed to identify genes and pathways that are immediately and lately shifted due to decreased and increased incubation temperature at two phases of myogenesis. Temperature adjustments at specific developmental time points could influence muscle development and potentially impact commercial meat production. In fact, modulation of *in-ovo* development with impact on post-hatch growth has been demonstrated in a number of experiments, which differ in terms of direction and extent of temperature change and the time period. Hammond et al. (2007) [18] and Lourens et al. (2005) [19] showed that higher incubation temperatures during the first week of *in-ovo* development increases total embryo mass; however no effect on post-hatch was found [19]. We previously reported that the exact conditions used here, i.e. high temperature (38.8°C) between ED 7 to 10 or ED 10 to 13 results in higher body weights of 35 d old broilers compared to broilers from normal (37.8°C) or lower (36.8°C) temperature conditions within these periods [20]. Increment of body weight was attributable to increased size of breast muscle rather than hind muscle [15]. The treatment intervals coincide with secondary fiber development. Treatments may thus have stronger impact on the breast muscle than on the hind muscle with the former mainly consisting of type II fibers originated from secondary fibers and the later mainly consisting of type I fibers originated from earlier developing primary fibers. Moreover, ED10 and ED13 embryos of the low temperature group had slightly but significantly lower weights than the control group at the respective time points; higher incubation temperature slightly but non-significantly increased the embryo weight (S3 Fig) [21]. Mitochondrial respiratory activity was lower in low temperature group at ED10 and ED13; higher incubation temperature led to higher mitochondrial respiratory rates at ED13. For metabolic enzymes only subtle mostly non-significant effects were found at the embryonic stages [21]. Accordingly, the D35 chickens analyzed here showed slight but significant increases in body or carcass weight when transiently incubated at higher temperature at ED7-10 or ED 10–13, whereas decreased incubation temperature did not affect either body weight or carcass weight (S4 Fig). Breast muscle weight of the 35 days old broilers was highest in the H10 group and lowest in the L13 groups. While these two extremes differed significantly, there were no significant differences among the other groups and no deviation from the respective controls.

The results suggest that transcriptional regulation taking place immediately *in-ovo* and in long-term after hatch displays mechanism that mediate resilient coping with low incubation temperature, whereas higher incubation temperature provokes phenotypic plasticity. We suspect that resilience to low temperature has been evolved by natural selection in bird species and still exists in commercial broiler lines. The phenotypic plasticity associated with higher incubation temperature offers perspectives for targeted modulation of traits relevant in poultry production by modulating incubation temperature.

### Incubation Temperature Has Immediate Effects on Gene Transcription at Embryonic Stages

Incubation temperature changes at both stages of myogenesis (ED7-10 and ED10-13) result in immediate changes to breast muscle gene transcription. In particular, higher incubation temperature during early myogenesis and lower temperature during later myogenesis promote the upregulation of many transcripts. The expression pattern of ED10 and ED13 were distinct, indicating the developmental stage has a larger effect than incubation temperature.

### Immediate Response to Early Modulation of Incubation Temperature

For early treatment, high temperature (H10ΔC) activated pathways in cell maintenance and organismal development, and especially affected survivability pathways. In contrast, low temperature (L10ΔC) down-regulated the differentiation and formation of muscle cells. The latter may contribute to the slightly lower body weight of ED10 embryos in the low temperature group. But since also liver and heart weight were significantly reduced in L10 compared to C10, low temperature seems to have a general quietening effect on development, not specific to myogenesis. Higher temperature did not provoke significant differences of weights (body, liver, and heart) compared to ED10 control embryos. Moreover, activity of mitochondrial respiration (state-3-pyruvate/malate and state-3-succinate/rotenone) and enzyme activities (glycogen phosphorylase, lactate dehydrogenase, and cytochrome oxidase) was shifted by low and high temperature with inhibiting and activating effects on metabolic processes, respectively [21]. The high breast muscle weight of D35 broilers of the H10 group is in line with the increased expression of genes of proliferative pathways, particularly at the early hyperplastic phase of secondary fibers formation, that become white fibers, the major proportion of fibers in the M. pectoralis. The up-regulated transcripts following high temperature treatment formed a network in tissue development and connective tissue and skeletal muscle system development and function. SMAD3 is the hub gene in that network. SMAD3 is a transcription factor involved in the regulation of growth factor expression including transforming growth factor and connective tissue growth factors that are relevant to many developmental processes including myogenesis. The regulatory effect in myoblast was shown recently [22]. Moreover, SMAD 3 is involved in myostatin signaling during myogenesis. Analyzes of differential expression of wild and myostatin knockout mice revealed that many DEG exhibited an SMAD3 binding motif [23]. In our study SMAD3 was down-regulated in muscle growth promoting hypertrophic conditions.

### Immediate Response to Late Modulation of Incubation Temperature

During later myogenesis (ED10-13), lower temperature produced more transcriptomic changes than did higher temperature. These conditions up-regulated pathways related to organismal survival and post-translational modification. Further prediction via Z-score revealed a potential prevention of organismal death and an increase in development of cardiovascular system, size of body, and metabolism of carbohydrate but an inactivation of cellular proliferation and differentiation. In result L13ΔC treatment is associated with reduced *in-ovo* growth, but has no impact on adult body weight. Higher temperature late treatment affected a few pathways. The possibility of regulation along this process tended to inhibit cytoplasm development and formation of cytoskeleton and filaments. In the L13ΔC network, pathways affecting cardiovascular system, skeletal-muscular, and connective tissue development are interconnected via RUNX2. RUNX2 plays a central role in osteoblastic differentiation and skeletal morphogenesis [24]. In chicken embryos, overexpression of RUNX2 produces multiple phenotypes including joint fusions, expansion of carpal elements, and shortening of skeletal elements [24]. On the other hand, inactivation of RUNX2 results in a disruption in chondrocyte differentiation, vascular invasion, osteoclast differentiation, and periosteal bone formation as seen by severe shortening of the limbs [24]. Downregulation of RUNX2 under low temperature conditions might suppress myogenesis process and embryo differentiation. This is in line with the reduced embryo body weight compared to control as well as enzyme activity (cytochrome oxidase) and mitochondrial respiration (state-3-pyruvate/malate) in the L13 group [21].

### Incubation Temperature Has Long-Term Effects on Gene Transcription at D35

*In-ovo* temperature manipulation also produced long-term transcriptional changes, as demonstrated by differential gene expression in adult chickens. Our findings add support to previous research that demonstrated that increased and/or decreased incubation temperature affects post-hatch growth in avian species [1,25,26,27,28].

### Long-Term Response to Early Modulation of Incubation Temperature

In adults, the highest number of genes with different transcript abundance compared to control was induced following low-temperature incubations at ED7-10 (L10ΔC). However, this did not lead to significant changes in either body weight or breast muscle weight of 35 day old broilers. Up- and down-regulated DEGs were related to cell cycle and nucleic acid processing with Z-scores indicating activation and deactivation, respectively. The DEG revealed a network related to gene expression, cellular function, and cell signaling pathways and covering genes belonging to the RNA polymerase II apparatus like MED14, MED15, MED23, MED24, TBP, and TRRAP along with this treatment. Increased incubation temperature at ED7-10 did not lead to shifts of expression in broilers that reveal any prominent pathways in terms of significance and Z-scores. MED24, a mediator or transcriptional coactivator, interacts with RNA polymerase II and promotes formation of a transcriptional pre-initiation complex [29]. Further, several related genes, like MED10, MED14, MED15, and MED23, were represented in the network. Downregulation of MED24 may alter RNA polymerase II activity and lead to abnormal transcription/translation of genes. Similarly, TBP (TATA-binding protein) interacts with transcription factor IID (TFIID), which binds to the core promoter to position RNA polymerase II properly [30]). Two forms of TBP mRNA are expressed in chicken [31]. Disruption of TBP causes phenotypic abnormalities with delayed mitosis and induced apoptosis [32]. Finally, TRRAP is a phosphoinositide 3-kinase-related kinase (PIKK) family member involved in transcription and DNA repair [29]. TRRAP is essential for early development, particularly for the mitotic checkpoint and regular cell cycle progression [33]. Thus, downregulation of genes following L10ΔC treatment may affect global gene transcription *in-ovo*.

### Long-Term Response to Late Modulation of Incubation Temperature

High temperature treatment during ED10-13 tended to suppress tissue development pathways, especially body size. Furthermore, nutrition metabolism, quantity, and synthesis of carbohydrates and lipids were also suppressed. In contrast, low-temperature treatment during ED10-13 tended to activate transcripts of trophic pathways and function while pathways related to cell death and apoptosis were reduced. In the nutrition group, metabolism of lipids was activated, but fat accumulation was deactivated, which may reduce concentrations of fatty acid components. In the network analysis, activation of organ, embryonic and skeletal-muscular system development were significant pathways with RAF1 and actin being hub genes. Actin represents an abundant protein with fundamental function in muscle tissue and the abundance of its transcript is linked with molecules that are not differentially expressed. Myosins are actin-based motor proteins that function in skeletal muscle contraction [34]. The proper function of both myosin heavy chain (MYH2) and myosin light chain (MYL3) are necessary to accompany actin filaments during eukaryotic motility processes [29]. Moreover, myogenin (MYOG), a muscle-specific transcription factor, is essential for developing functional skeletal muscle [35]. In chicken, MYOG affects muscle fiber trait specification [36]. Thus, upregulation of these genes could enhance muscle cell and contraction processes. RAF1, which links also T-actin, is a MAP3K, i.e. a higher order kinase affecting the ERK-pathway via MEK1 and MEK2. The serine/threonine specific protein kinases, ERK1 and ERK2 are involved in control of gene expression and by this have effects on cell and tissue formation. Only recently it was shown in mice that the ERK1/2 pathways is essential for the maintenance of adult muscle fibers and the link of the nerval and muscle system [37]. Also in chicken the ERK1/2 MAP is known to be involved in protein synthesis pathways particularly in myoblast cells [38]. Modulated incubation temperatures had significant effects on adult transcriptomes, and led to subtle but still significant phenotypic differences as long as increased temperature is concerned, i.e. increased body and breast muscle weight due to high incubation temperature. Lowered incubation temperature led to shifts of expression of an even higher number of genes but was not associated with significant phenotypic changes of day 35 broilers.

## Conclusion

Increasing as well as decreasing of incubation temperature at two stages (ED7-10 and ED10-13) affected the abundance of numerous transcript immediately and in the long-term. Functional annotation indicated that these genes are assigned to biofunctions related to cell formation and survival tending to be promoted, whereas metabolic pathways were less modulated. However, the sets of modulated genes were mostly specific to the different treatments. The fact that increased incubation temperature increased organismal growth but lowered temperature did not affect the phenotype at D35 suggest that the shifts of expression associated with low temperature represent molecular routes promoting resilience to the treatment. In contrast, elevated incubation temperature conditions the organisms for increased growth. The altered expression displays the molecular pathways that mediate the phenotypic plasticity. The results have implications in terms of natural selection and the development of mechanisms to cope with advise conditions and in terms of deriving strategies to improve poultry breeding. Epigenetic temperature acclimatization might alter body growth and enrich poultry resistance to various environmental effects. It would be important to address the epigenetic changes at the molecular level in future studies.

## Supporting Information

S1 Fig IPA legend of network shapes and relationships. (source http://ingenuity.force.com/ipa/articles/Feature_Description/Legend).
(TIF)

S2 Fig Major gene networks derived from genes consistently regulated at embryonic stages and D35. For L10ΔC a network related to cell-to-cell signaling and interaction, nervous system development, and cell survival pathway was derived (A). For L13ΔC a network related to cell death and survival, cellular development, and cellular growth and proliferation was derived (B). Red color, up-regulated; Green color, down-regulated.
(TIF)

S3 Fig Body weight after exposure to H (High 38.8°C), C (Control 37.8°C), and L (Low 36.8°C) temperature at the end of intervention, ED10 and ED13, respectively (adapt from [17]). (DOCX)

S4 Fig Body, carcass and breast muscle weights at day 35 after *in-ovo* exposure to H (High 38.8°C), C (Control 37.8°C), and L (Low 36.8°C) temperature at ED7-10 and ED10-13. (DOCX)

S1 Table Numbers of differentially expressed probes sets at p ≤ 0.05 and corresponding q-value for the variance components. The comparisons between each *in-ovo* thermal modification condition and the time-matched control separated for embryonic stages or post-hatch D35.
(DOCX)

S2 Table DEGs common to both embryonic stages and D35. (DOCX)

S3 Table Assignment of DEGs to biological functions (major categories and Ingenuity-biofunctions) (*p*≤0.05) obtained at embryonic stage for early treatment; H10UΔC, H10DΔC, L10UΔC and L10DΔC. (DOCX)

S4 Table Assignment of DEGs to biological functions (major categories and Ingenuity-biofunctions) (*p*≤0.05) obtained at embryonic stage for late treatment; H13UΔC, H13DΔC, L13UΔC and L13DΔC. (DOCX)

S5 Table Assignment of DEGs to biological functions (major categories and Ingenuity-biofunctions) (*p*≤0.05) obtained at adult stage for early treatment; H10UΔC, H10DΔC, L10UΔC and L10DΔC. (DOCX)

S6 Table Assignment of DEGs to biological functions (major categories and Ingenuity-biofunctions) (*p*≤0.05) obtained at adult stage for late treatment; H13UΔC, H13DΔC, L13UΔC and L13DΔC. (DOCX)
