# Supplementary figures and images for: Transient Shifts of Incubation Temperature Reveal Immediate and Long-Term Transcriptional Response in Chicken Breast Muscle Underpinning Resilience and Phenotypic Plasticity
Source: PLoS One. 2016 Sep 9;11(9):e0162485. doi: 10.1371/journal.pone.0162485 (PMC5017601; doi:10.1371/journal.pone.0162485)

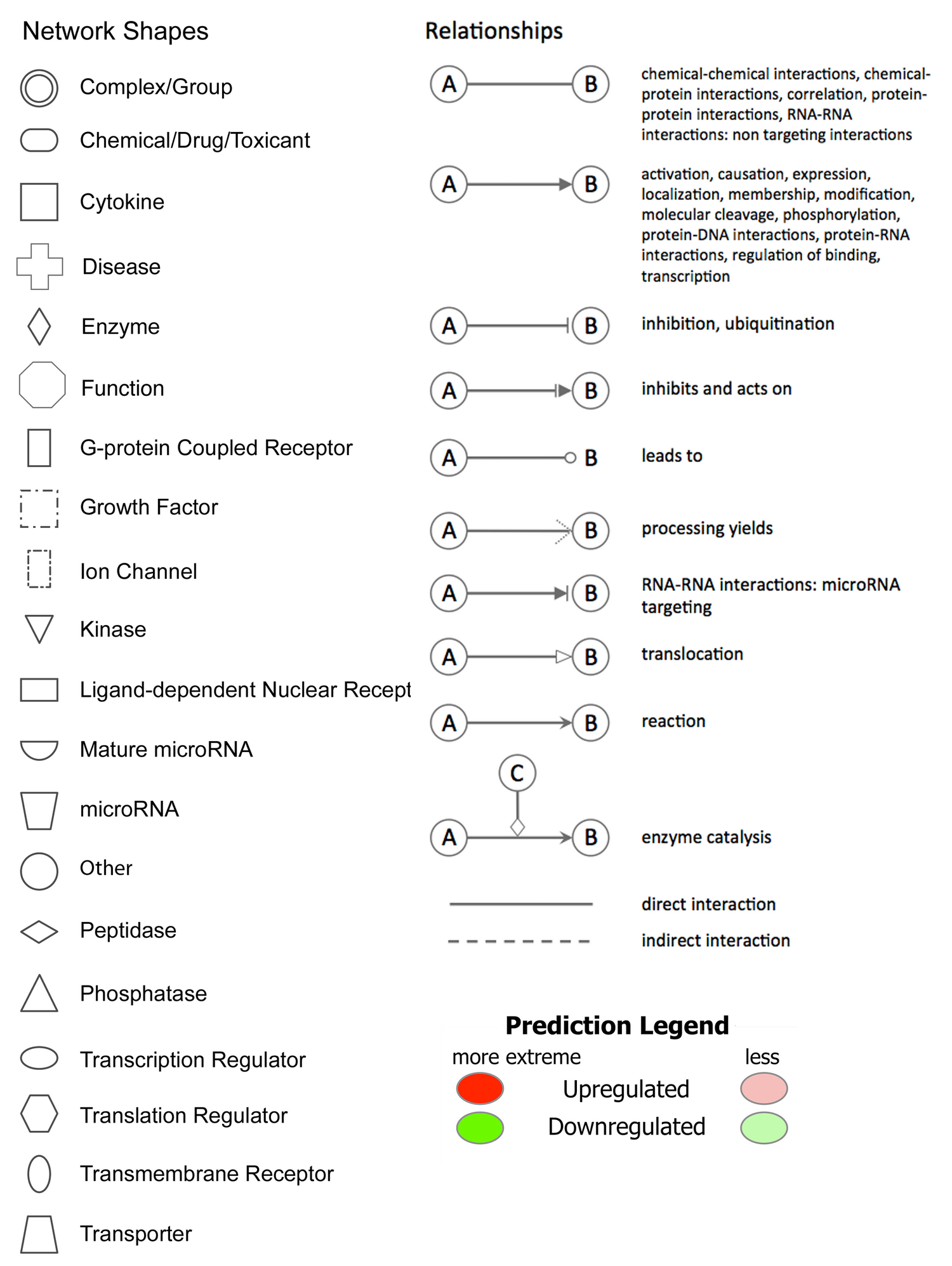

Supplement: S1 Fig — (source http://ingenuity.force.com/ipa/articles/Feature_Description/Legend). (TIF) [file pone.0162485.s001.tif]

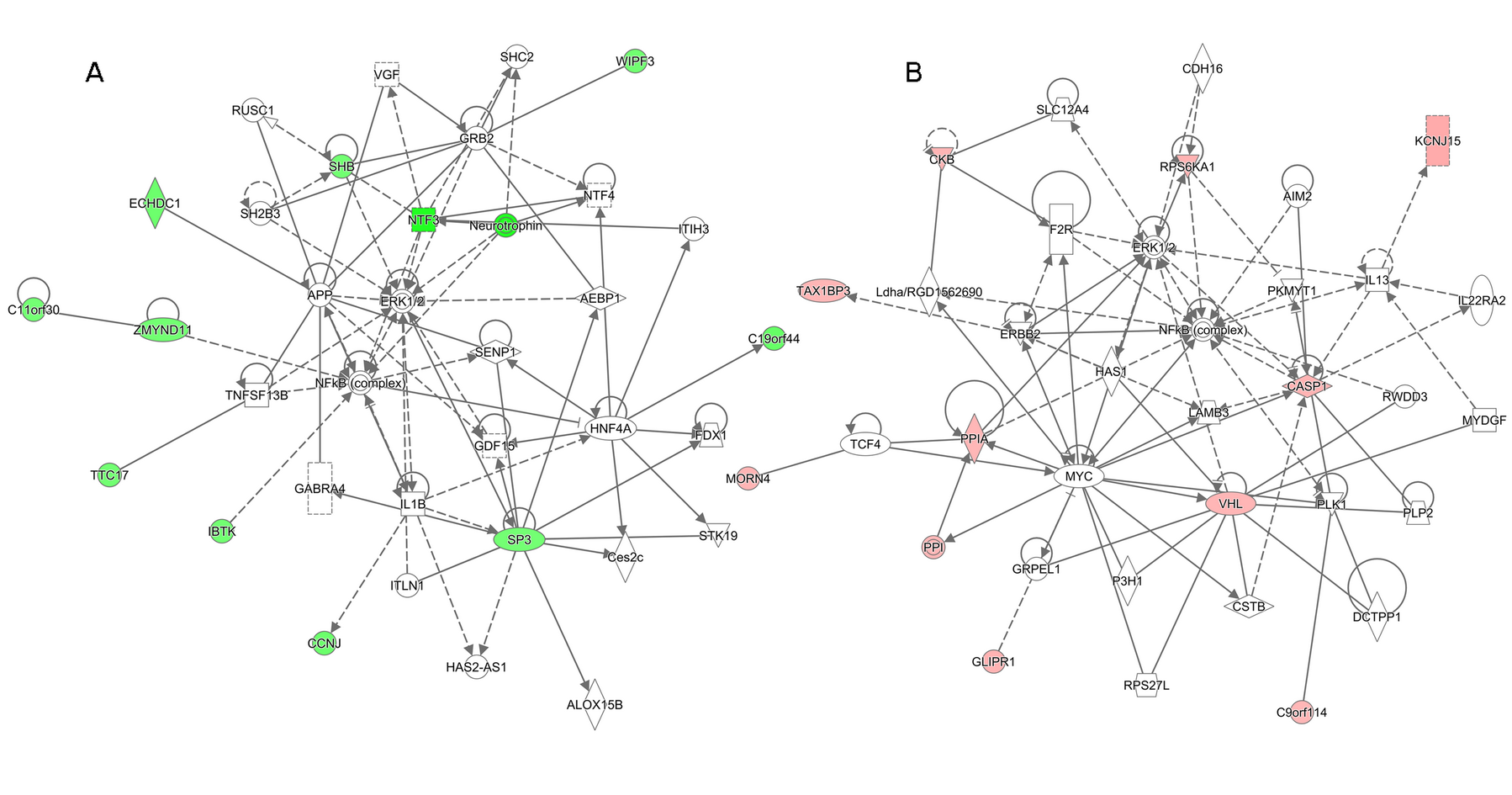

Supplement: S2 Fig — For L10ΔC a network related to cell-to-cell signaling and interaction, nervous system development, and cell survival pathway was derived (A). For L13ΔC a network related to cell death and survival, cellular development, and cellular growth and proliferation was derived (B). Red color, up-regulated; Green color, down-regulated. (TIF) [file pone.0162485.s002.tif]
