# Supplementary material for: Transient Shifts of Incubation Temperature Reveal Immediate and Long-Term Transcriptional Response in Chicken Breast Muscle Underpinning Resilience and Phenotypic Plasticity
Source: PLoS One. 2016 Sep 9;11(9):e0162485. doi: 10.1371/journal.pone.0162485 (PMC5017601; doi:10.1371/journal.pone.0162485)
Supplement: S3 Fig — (DOCX) [file pone.0162485.s003.docx]

**0**

**2**

**4**

**6**

**8**

**10**

**12**

**Control ED 10**

**ED7-10-H**

**ED 7-10-L**

**Control ED 13**

**ED 10-13-H**

**ED 10-13-L**

**Weights in gram**

**c**

**c**

**d**

**a**

**a**

**b**

**c**

**c**

**d**

**a**

**a**

**b**

**Body weight at ED7-10 and ED10-13**

**S3 Fig. Phenotypic results on body weight during incubation stage.** Body weight changes after expose to H (High 38.8 °C), C (Control 37.8 °C), and L (Low 36.8 °C) temperature at the end of temperature intervention between ED (Embryonic day) 7-10 and ED10-13 treatment (adapt from [17]).
