# Supplementary material for: Transient Shifts of Incubation Temperature Reveal Immediate and Long-Term Transcriptional Response in Chicken Breast Muscle Underpinning Resilience and Phenotypic Plasticity
Source: PLoS One. 2016 Sep 9;11(9):e0162485. doi: 10.1371/journal.pone.0162485 (PMC5017601; doi:10.1371/journal.pone.0162485)
Supplement: S4 Fig — (DOCX) [file pone.0162485.s004.docx]

**S4 Fig. Phenotypic result on body, carcass and both breast weight at day 35.** H, high temperature; L, low temperature; C, control; 10, ED10 was the end of temperature intervention; 13, ED13 was the end of temperature intervention.
