# Supplementary material for: Transient Shifts of Incubation Temperature Reveal Immediate and Long-Term Transcriptional Response in Chicken Breast Muscle Underpinning Resilience and Phenotypic Plasticity
Source: PLoS One. 2016 Sep 9;11(9):e0162485. doi: 10.1371/journal.pone.0162485 (PMC5017601; doi:10.1371/journal.pone.0162485)
Supplement: S1 Table — The comparisons between each in-ovo thermal modification condition and the time-matched control separated for embryonic stages or post-hatch D35. (DOCX) [file pone.0162485.s005.docx]

**S1 Table. Numbers of differentially expressed probes sets at p ≤ 0.05 and corresponding q-value for the variance components.** The comparisons between each *in-ovo* thermal modification condition and the time-matched control separated for embryonic stages or post-hatch D35*.*

|  | **Treatment** | **q-value** | **Probe-sets** |
| --- | --- | --- | --- |
|  | Fix effect: Temperature (H,C,L) | 0.1435 | 2896 |
|  | Fix effect: Date (ED7-10, ED10-13) | 0.0775 | 5370 |
|  | H10ΔC | 0.1414 | 1484 |
| Embryo | H13ΔC | 0.1414 | 470 |
|  | L10ΔC | 0.1414 | 415 |
|  | L13ΔC | 0.1412 | 905 |
|  | Fix effect: Temperature (H,C,L) | 0.3327 | 1250 |
|  | Fix effect: Date (ED7-10, ED10-13) | 0.6754 | 616 |
|  | Co-effect: Breast weight | 0.1852 | 2245 |
| D35 | H10ΔC | 0.6441 | 262 |
|  | H13ΔC | 0.6441 | 325 |
|  | L10ΔC | 0.6441 | 846 |
|  | L13ΔC | 0.6441 | 349 |
