# Supplementary material for: Transient Shifts of Incubation Temperature Reveal Immediate and Long-Term Transcriptional Response in Chicken Breast Muscle Underpinning Resilience and Phenotypic Plasticity
Source: PLoS One. 2016 Sep 9;11(9):e0162485. doi: 10.1371/journal.pone.0162485 (PMC5017601; doi:10.1371/journal.pone.0162485)
Supplement: S2 Table — (DOCX) [file pone.0162485.s006.docx]

**S1 Table. DEGs common to both embryonic stages and D35.**

| **Treatment Group** | **Transcript ID** | **Gene symbol** | **Embryo** | | | **Adult** | | |
| --- | --- | --- | --- | --- | --- | --- | --- | --- |
|  |  |  | **P-value** | **FC** | **FC trend** | **P-value** | **FC** | **FC trend** |
| **H10** | 15388467 | *AKR1D1* | 0.0012 | -1.31975 | DOWN | 0.042 | -1.17708 | DOWN |
|  | 15388518 | *DKFZp547G036* | 0.023 | -1.16486 | DOWN | 0.005 | -1.10758 | DOWN |
|  | 15393184 | *HDHD1A* | 0.013 | 1.334102 | UP | 0.0487 | -1.04332 | DOWN |
|  | 15399264 | *AT2B1_CHICK* | 0.0219 | 1.308808 | UP | 0.0379 | -1.07543 | DOWN |
|  | 15405704 | *CREG2* | 0.0344 | 1.331552 | UP | 0.0011 | -1.26069 | DOWN |
|  | 15406397 | *MIR18A* | 0.0051 | 1.732387 | UP | 0.0029 | -21.7465 | DOWN |
|  | 15409988 | *TEX9* | 0.0314 | 1.42904 | UP | 0.016 | -1.20225 | DOWN |
|  | 15411964 | *SCAMP5* | 0.0009 | -1.16402 | DOWN | 0.0343 | -1.0567 | DOWN |
|  | 15413660 | *TIPIN_CHICK* | 0.0071 | 1.211334 | UP | 0.03 | -1.09928 | DOWN |
|  | 15415679 | *ARYL_CHICK* | 0.0094 | 1.712505 | UP | 0.0147 | -1.14876 | DOWN |
|  | 15417679 | *CES1* | 0.0422 | 1.6867 | UP | 0.0341 | -1.47318 | DOWN |
|  | 15420113 | *C3orf14* | 0.0289 | 1.220518 | UP | 0.0173 | -1.11951 | DOWN |
|  | 15421706 | *Q5ZKS9_CHICK* | 0.0119 | 1.133635 | UP | 0.0468 | -1.0263 | DOWN |
|  | 15426187 | *SPRY4* | 0.0442 | -1.19905 | DOWN | 0.0219 | -1.4395 | DOWN |
|  | 15429749 | *SHMT1* | 0.0083 | 1.629462 | UP | 0.0164 | -1.16007 | DOWN |
|  | 15441057 | *NOGG_CHICK* | 0.0207 | -1.16031 | DOWN | 0.0277 | 1.136548 | UP |
|  | 15444046 | *CALN1* | 0.0159 | -1.27491 | DOWN | 0.0238 | -4.06126 | DOWN |
|  | 15444897 | *CCDC55* | 0.0009 | 1.713538 | UP | 0.0004 | -1.30275 | DOWN |
|  | 15449899 | *TSPAN13* | 0.0105 | 1.516165 | UP | 0.0301 | -1.04443 | DOWN |
|  | 15454357 | *CRLD1_CHICK* | 0.0376 | 1.325579 | UP | 0.0217 | -1.2754 | DOWN |
|  | 15455729 | *GIMAP8* | 0.0157 | 1.394909 | UP | 0.0186 | -1.77575 | DOWN |
|  | 15475100 | *CCDC153* | 0.0158 | -1.15652 | DOWN | 0.0028 | 1.141955 | UP |
|  | 15487096 | *Q5UKY7_CHICK* | 0.0087 | 1.349626 | UP | 0.046 | -1.05974 | DOWN |
|  | 15490320 | *DDO* | 0.0014 | 1.648523 | UP | 0.0335 | -1.09344 | DOWN |
|  | 15497510 | *SNORD101* | 0.018 | 1.287028 | UP | 0.0299 | 1.491588 | UP |
|  | 15499195 | *ADI1* | 0.0153 | 1.252039 | UP | 0.0193 | -1.04217 | DOWN |
|  | 15503912 | *HSD17B11* | 0.0454 | 1.310789 | UP | 0.0376 | -1.09713 | DOWN |
|  | 15505078 | *GgaAffx.8719.1.S1_at* | 0.0468 | 1.249491 | UP | 0.0444 | -1.06882 | DOWN |
|  | 15505748 | *Q60GU0_CHICK* | 0.0002 | 1.533635 | UP | 0.0043 | -1.09463 | DOWN |
|  | 15506087 | *KIAA0232* | 0.0204 | 1.305299 | UP | 0.0299 | -1.05135 | DOWN |
|  | 15511713 | *Q5ZLP7_CHICK* | 0.03 | 1.381465 | UP | 0.0243 | -1.08619 | DOWN |
|  | 15517174 | *TMEM63C* | 0.0342 | -1.1498 | DOWN | 0.0137 | -1.06944 | DOWN |
|  | 15517860 | *ZNF839* | 0.0351 | 1.300379 | UP | 0.0231 | -1.10352 | DOWN |
|  | 15533588 | *CHPF* | 0.0081 | -1.20758 | DOWN | 0.0451 | -1.10051 | DOWN |
|  | 15541032 | *HOOK1_CHICK* | 0.0178 | 1.574125 | UP | 0.0092 | -1.132 | DOWN |
|  | 15545357 | *KLHL24* | 0.0056 | 1.178896 | UP | 0.0084 | -1.08576 | DOWN |
|  | 15551431 | *MYH11* | 0.0035 | 2.226002 | UP | 0.0372 | -1.09346 | DOWN |
|  | 15554125 | *Gga.8084.2.S1_a_at* | 0.0402 | 1.742633 | UP | 0.01 | -26.626 | DOWN |
|  | 15556371 | *FFAR2* | 0.0017 | -1.32583 | DOWN | 0.0475 | -1.31134 | DOWN |
|  | 15558305 | *NLN* | 0.0328 | 1.335125 | UP | 0.0458 | -1.14565 | DOWN |
|  | 15560998 | *MCCC2* | 0.0134 | 1.406738 | UP | 0.0267 | -1.10782 | DOWN |
|  | 15566030 | *gga-mir-1594* | 0.0456 | -1.18453 | DOWN | 0.005 | 1.148234 | UP |
| **L10** | 15389859 | *NTF3* | 0.0435 | -1.30129 | DOWN | 0.0124 | -1.67295 | DOWN |
|  | 15390556 | *KIAA2018* | 0.0462 | -1.1774 | DOWN | 0.0481 | -1.05696 | DOWN |
|  | 15395399 | *MB211_CHICK* | 0.0112 | 1.187371 | UP | 0.0341 | -1.32955 | DOWN |
|  | 15399245 | *DUSP6* | 0.0428 | 1.11886 | UP | 0.048 | -1.05343 | DOWN |
|  | 15399264 | *AT2B1_CHICK* | 0.0381 | -1.2739 | DOWN | 0.0069 | -1.10221 | DOWN |
|  | 15401885 | *Q5ZKY8_CHICK* | 0.0487 | -1.44133 | DOWN | 0.0044 | -1.11051 | DOWN |
|  | 15408536 | *C11orf30* | 0.0449 | -1.12767 | DOWN | 0.0182 | -1.03636 | DOWN |
|  | 15411964 | *SCAMP5* | 0.0463 | 1.090877 | UP | 0.0328 | -1.05676 | DOWN |
|  | 15418759 | *TMEM111* | 0.0428 | 1.216625 | UP | 0.0205 | -1.09206 | DOWN |
|  | 15421704 | *KLHDC6* | 0.0451 | -1.40915 | DOWN | 0.0041 | -1.07391 | DOWN |
|  | 15435883 | *MTFP1* | 0.0469 | 1.226265 | UP | 0.018 | -1.11739 | DOWN |
|  | 15445566 | *TAD2A_CHICK* | 0.0455 | 1.090546 | UP | 0.0028 | -1.05584 | DOWN |
|  | 15448679 | *ZMYND11* | 0.0494 | -1.29068 | DOWN | 0.0037 | -1.05093 | DOWN |
|  | 15450183 | *WIPF3* | 0.0023 | -1.25382 | DOWN | 0.0095 | -1.06351 | DOWN |
|  | 15462917 | *C8orf83* | 0.0033 | -1.36136 | DOWN | 0.0266 | -1.13792 | DOWN |
|  | 15480577 | *DAD1_CHICK* | 0.0496 | 1.068228 | UP | 0.0162 | -1.02738 | DOWN |
|  | 15485015 | *C19orf44* | 0.0182 | -1.36009 | DOWN | 0.0324 | -1.143 | DOWN |
|  | 15489907 | *ECHDC1* | 0.0373 | -1.26077 | DOWN | 0.0483 | -1.09067 | DOWN |
|  | 15491144 | *IBTK* | 0.0336 | -1.33425 | DOWN | 0.0181 | -1.05229 | DOWN |
|  | 15498961 | *TMEM14A* | 0.0418 | 1.150348 | UP | 0.0279 | -1.0839 | DOWN |
|  | 15499582 | *Q2VB18_CHICK* | 0.0473 | -1.08735 | DOWN | 0.0336 | -1.03541 | DOWN |
|  | 15513754 | *Q5ZLZ4_CHICK* | 0.0419 | 1.116244 | UP | 0.0088 | -1.04802 | DOWN |
|  | 15525105 | *FAM149B1* | 0.0337 | -1.1359 | DOWN | 0.0375 | -1.04543 | DOWN |
|  | 15526614 | *TBC1D12* | 0.025 | -1.23289 | DOWN | 0.0349 | -1.06191 | DOWN |
|  | 15530154 | *CCNJ* | 0.0431 | -1.18824 | DOWN | 0.0403 | -1.09813 | DOWN |
|  | 15532933 | *SP3_CHICK* | 0.0231 | -1.23068 | DOWN | 0.0298 | -1.04104 | DOWN |
|  | 15535607 | *SDPR* | 0.0172 | -1.18256 | DOWN | 0.025 | -1.03555 | DOWN |
|  | 15535718 | *C2orf66* | 0.0119 | -1.4506 | DOWN | 0.0198 | -1.15849 | DOWN |
|  | 15542125 | *ITPA* | 0.0105 | 1.195942 | UP | 0.0355 | -1.0888 | DOWN |
|  | 15549507 | *Q5ZHL1_CHICK* | 0.0002 | 1.187535 | UP | 0.0329 | -1.09436 | DOWN |
|  | 15551140 | *AGXT2L1* | 0.0369 | -1.54604 | DOWN | 0.045 | 1.140921 | UP |
|  | 15554171 | *TTC17* | 0.0394 | -1.2451 | DOWN | 0.0377 | -1.05221 | DOWN |
|  | 15554202 | *MANSC1* | 0.0319 | -1.3761 | DOWN | 0.0073 | -1.07229 | DOWN |
|  | 15559926 | *SHB* | 0.0384 | -1.18887 | DOWN | 0.0389 | -1.05387 | DOWN |
| **H13** | 15384402 | *PODXL_CHICK* | 0.0452 | 1.121739 | UP | 0.0349 | 1.021131 | UP |
|  | 15392667 | *TMEM47* | 0.0484 | 1.167657 | UP | 0.0367 | 1.032571 | UP |
|  | 15403705 | *RN5-8S1* | 0.0047 | -1.2424 | DOWN | 0.0287 | -1.02403 | DOWN |
|  | 15419250 | *FGD3* | 0.0168 | -1.14818 | DOWN | 0.039 | -1.24181 | DOWN |
|  | 15434604 | *Q5ZKS2_CHICK* | 0.046 | 1.282928 | UP | 0.0344 | -1.0958 | DOWN |
|  | 15436224 | *CLEC2B* | 0.001 | 5.27647 | UP | 0.0088 | 1.174525 | UP |
|  | 15436422 | *CLEC2B* | 0.0307 | 3.752219 | UP | 0.0331 | 1.332476 | UP |
|  | 15436477 | *MR1* | 0.009 | 2.816325 | UP | 0.0149 | 1.195907 | UP |
|  | 15436481 | *SBF1* | 0.0181 | 1.994104 | UP | 0.0222 | 1.05072 | UP |
|  | 15439748 | *FAM69B* | 0.0466 | 1.161778 | UP | 0.0325 | -1.10215 | DOWN |
|  | 15452962 | *NETO1* | 0.0241 | 1.750302 | UP | 0.0401 | -1.38743 | DOWN |
|  | 15459552 | *KCNG2_CHICK* | 0.0151 | 1.278274 | UP | 0.0493 | -1.08942 | DOWN |
|  | 15474103 | *ZC3H12A* | 0.0093 | 1.19145 | UP | 0.0235 | 1.03375 | UP |
|  | 15483075 | *SNORD37* | 0.0055 | -1.69955 | DOWN | 0.0046 | -1.16873 | DOWN |
|  | 15488159 | *AKT3* | 0.0333 | 1.396074 | UP | 0.0459 | 1.125438 | UP |
|  | 15496847 | *Q5ZHQ4_CHICK* | 0.0193 | -1.1689 | DOWN | 0.0413 | -1.07181 | DOWN |
|  | 15509009 | *IL15* | 0.0056 | 1.457787 | UP | 0.0411 | -1.08185 | DOWN |
|  | 15540985 | *Q2LAI0_CHICK* | 0.0492 | 1.178853 | UP | 0.0275 | 1.024463 | UP |
|  | 15549507 | *Q5ZHL1_CHICK* | 0.0412 | -1.09188 | DOWN | 0.0407 | 1.06123 | UP |
|  | 15551255 | *RN5-8S1* | 0.0047 | -1.2424 | DOWN | 0.0287 | -1.02403 | DOWN |
|  | 15552380 | *BAHCC1* | 0.0082 | 1.143686 | UP | 0.0443 | -1.04393 | DOWN |
|  | 15566038 | *Q9PRY6_CHICK* | 0.0249 | -1.23749 | DOWN | 0.0423 | -1.02504 | DOWN |
| **L13** | 15386501 | *Q5F3D6_CHICK* | 0.0241 | 1.434946 | UP | 0.048 | 1.084467 | UP |
|  | 15392159 | *KCNJ15* | 0.0405 | 1.241699 | UP | 0.0466 | 1.196927 | UP |
|  | 15400236 | *Q5F377_CHICK* | 0.0168 | 1.16958 | UP | 0.0191 | 1.040803 | UP |
|  | 15402320 | *RM51_CHICK* | 0.0355 | 1.124779 | UP | 0.0267 | -1.02967 | DOWN |
|  | 15418520 | *GgaAffx.1460.1.S1_at*  *(CACNA2D2)* | 0.0277 | 1.10474 | UP | 0.0188 | 1.029874 | UP |
|  | 15420471 | *VHL* | 0.0044 | 1.176338 | UP | 0.0449 | 1.038437 | UP |
|  | 15431039 | *FAM86A* | 0.0189 | 1.450101 | UP | 0.0456 | 1.089673 | UP |
|  | 15431766 | *TMEM132D* | 0.0478 | 1.231661 | UP | 0.014 | -1.43548 | DOWN |
|  | 15436224 | *CLEC2B* | 0.0295 | 2.890328 | UP | 0.0229 | 1.149088 | UP |
|  | 15439316 | *C9orf114* | 0.008 | 1.146318 | UP | 0.0459 | 1.02647 | UP |
|  | 15443987 | *O42284_CHICK (CASP1)* | 0.0194 | 1.152428 | UP | 0.0342 | 1.062649 | UP |
|  | 15445040 | *TAX1BP3* | 0.0347 | 1.088703 | UP | 0.0319 | 1.038566 | UP |
|  | 15447772 | *Q5ZHK3_CHICK* | 0.0132 | 1.16538 | UP | 0.0325 | -1.03171 | DOWN |
|  | 15455056 | *BAALC* | 0.0059 | -1.16954 | DOWN | 0.0042 | 1.14889 | UP |
|  | 15467861 | *NPEPL1* | 0.003 | 1.159972 | UP | 0.0282 | -1.01704 | DOWN |
|  | 15468560 | *C1orf70* | 0.0109 | 1.36351 | UP | 0.0245 | 2.916026 | UP |
|  | 15471254 | *LOXL2* | 0.0352 | 1.143961 | UP | 0.0213 | -1.05891 | DOWN |
|  | 15472806 | *FHL3* | 0.0418 | 1.124926 | UP | 0.0014 | 1.050148 | UP |
|  | 15473550 | *RPS6KA1* | 0.0002 | 1.24716 | UP | 0.0302 | 1.083721 | UP |
|  | 15479975 | *C1orf183* | 0.0471 | 1.261173 | UP | 0.0403 | 1.051062 | UP |
|  | 15515400 | *COMMD9* | 0.0101 | 1.184172 | UP | 0.0162 | 1.125434 | UP |
|  | 15519864 | *RS13_CHICK* | 0.0068 | 1.149144 | UP | 0.0105 | 1.0223 | UP |
|  | 15523718 | *KCRB_CHICK (CKB)* | 0.0038 | 1.366353 | UP | 0.0349 | 1.051569 | UP |
|  | 15526978 | *Q5ZK06_CHICK* | 0.0076 | 1.17598 | UP | 0.0413 | 1.050222 | UP |
|  | 15544277 | *TMEM48* | 0.0317 | 1.118612 | UP | 0.0406 | -1.04254 | DOWN |
|  | 15554600 | *PPIA* | 0.0093 | 1.134455 | UP | 0.0333 | 1.027108 | UP |
|  | 15554961 | *Q2L7E6_CHICK* | 0.0298 | 1.146374 | UP | 0.0442 | 1.045147 | UP |
|  | 15556735 | *FUBP3* | 0.0216 | 1.116526 | UP | 0.0112 | -1.05649 | DOWN |

FC, fold-chang
