# Supplementary material for: Transient Shifts of Incubation Temperature Reveal Immediate and Long-Term Transcriptional Response in Chicken Breast Muscle Underpinning Resilience and Phenotypic Plasticity
Source: PLoS One. 2016 Sep 9;11(9):e0162485. doi: 10.1371/journal.pone.0162485 (PMC5017601; doi:10.1371/journal.pone.0162485)
Supplement: S3 Table — (DOCX) [file pone.0162485.s007.docx]

**S3** **Table. Assignment of DEGs to biological functions (major categories and Ingenuity-biofunctions) (*p*≤0.05) obtained at embryonic stage for early treatment; H10UΔC, H10DΔC, L10UΔC and L10DΔC.**

| **Major categories** | **Ingenuity-biofunction** | | **BH p-value** | **Z-score** | **# of DEGs** | **DEGs assigned to biofunction*** | |
| --- | --- | --- | --- | --- | --- | --- | --- |
| **H10U** |  | | | | | | |
| **Cell maintenance, proliferation differentiation and replacement** | stress response of cells | | 1.83E-02 | -2.151 | 17 | AHR, BRCA1, CASP3, TSC22D3, UGT8, IGF1, PARK2, AKT1, ATRX, CASP8 | |
|  | apoptosis of testicular cells | | 1.07E-02 | -2 | 6 |  |  |
|  | necrosis of skeletal muscle cells | | 4.00E-02 | -1.929 | 4 |  |  |
|  | apoptosis of gonadal cells | | 1.30E-02 | -1.71 | 22 |  |  |
|  | apoptosis of germ cells | | 4.63E-02 | -1.343 | 16 |  |  |
|  | proliferation of fibroblast cell lines | | 2.15E-02 | 2.172 | 41 | AKT1, BRCA1, DICER1, EGFR, GRK5, IGF1, KLF5, PARK2, PIK3R1, RB1 | |
|  | microtubule dynamics | | 3.66E-02 | 2.281 | 55 |  |  |
|  | organization of cytoplasm | | 6.91E-03 | 2.368 | 84 |  |  |
|  | organization of cytoskeleton | | 1.87E-02 | 2.368 | 72 |  |  |
|  | proliferation of cells | | 4.66E-02 | 3.892 | 214 |  |  |
| **Organismal, organ and tissue development** | organismal death | | 1.93E-05 | -12.362 | 200 | PSEN1, ACVR2B, AKT1, ARHGAP5, ARID4B, BRCA1, CDK6, CEBPA, IGF1, MAN2A1 | |
|  | perinatal death | | 1.87E-02 | -7.214 | 54 |  |  |
|  | morphology of body cavity | | 4.00E-02 | -2.596 | 87 |  |  |
|  | morphology of lymphatic system component | | 3.62E-02 | -2.2 | 37 |  |  |
|  | short-term memory | | 1.81E-02 | -0.152 | 7 |  |  |
|  | quantity of corpus luteum | | 3.95E-02 | 1.89 | 7 | AHR, IGF1, SOD1, CEBPB, GHR, AKT1, BCL6, BRCA1, CDK6, CREB1 | |
|  | survival of organism | | 3.22E-02 | 3.09 | 60 |  |  |
|  | development of genital organ | | 2.75E-02 | 3.499 | 47 |  |  |
|  | gonadogenesis | | 3.02E-02 | 3.86 | 44 |  |  |
|  | size of body | | 4.22E-03 | 7.964 | 78 |  |  |
| **Nutrient metabolism** | concentration of acylglycerol | | 3.27E-02 | -0.071 | 34 | ACSL1, AHSG, AKT1, CEBPA, CEBPB, CES1, CIDEA, ECI1, EGFR, FAAH | |
|  | concentration of fatty acid | | 4.00E-02 | 0.137 | 28 | AKT1, CES1, GPAM, IGF1, PPARA, PPARGC1A, AHSG, CEBPA, FABP1, COMT | |
|  | concentration of triacylglycerol | | 4.42E-02 | 0.36 | 31 |  |  |
|  | metabolism of amino acids | | 2.15E-02 | 2.384 | 20 |  |  |
|  | metabolism of nucleic acid component or derivative | | 3.62E-02 | 3.049 | 55 |  |  |
| **Genetic information and nucleic acid processing** | DNA damage | | 1.81E-02 | -1.283 | 19 | AHR, BRCA1, CASP3, CASP8, CAT, DICER1, EGFR, EIF2AK2, IGF1, mir-15 | |
|  | synthesis of protein | | 4.70E-02 | 0.939 | 38 | AKT1, EGFR, IGF1, AHR, NPM1, EIF2AK2, PIK3R1, BRCA1, KITLG, POLA1 | |
|  | metabolism of DNA | | 1.46E-02 | 1.705 | 37 |  |  |
|  | DNA replication | | 1.87E-02 | 2.564 | 25 |  |  |
|  | phosphorylation of protein | | 4.00E-02 | 2.764 | 55 |  |  |
|  | repair of DNA | | 3.28E-02 | 2.864 | 24 |  |  |
| **Cell signaling and interaction** | Rho protein signal transduction | | 1.34E-02 |  | 12 | AGTR1, ARHGAP29, ARHGEF3, BCL6, CUL3, DLC1, MYO9B, PTPLAD1, ROCK1, SYNJ2BP | |
|  | insulin-like growth factor receptor signaling pathway | | 2.61E-02 |  | 5 |  |  |
|  | activation of cyclin-dependent protein kinase | | 4.00E-02 |  | 5 |  |  |
| **Small molecule biochemistry** | beta-oxidation of lipid | | 4.25E-03 | 1.72 | 14 | ABCD3, ACADSB, ACOX2, BDH2, CPT1A, CPT2, DECR1, ECHS1, FABP1, PPARA | |
|  | oxidation of lipid | | 2.45E-03 | 2.02 | 30 |  |  |
|  | oxidation of fatty acid | | 3.74E-03 | 2.327 | 24 |  |  |
|  | beta-oxidation of fatty acid | | 1.14E-02 | 2.355 | 13 |  |  |
| **H10D** |  |  | |  |  |  |  |
| **Cell maintenance, proliferation differentiation and replacement** | cell movement | | 6.98E-03 | -3.189 | 46 | CDKN1B, NFATC1, SMAD3, GSN, TPM1, CAV3, COL1A1, CORO1C, CYR61, FHL1 | |
|  | migration of cells | | 4.60E-03 | -3.114 | 44 |  |  |
|  | formation of cytoskeleton | | 4.84E-02 | -1.97 | 12 |  |  |
|  | formation of filaments | | 4.91E-02 | -1.863 | 13 |  |  |
|  | proliferation of connective tissue cells | | 4.20E-02 | -0.198 | 12 |  |  |
| **Organismal, organ and tissue development** | development of connective tissue | | 3.30E-02 | -1.219 | 12 | CYR61, NFATC1, PPARD, SMAD3, SOCS3, CHST3, CHSY1, CSPG4, FMOD, GSN | |
|  | growth of connective tissue | | 3.39E-02 | -0.142 | 13 |  |  |
|  | formation of muscle | | 4.75E-02 | 0.469 | 14 | CAV3, FMOD, FOXC1, HOXD10, KLF2, SMAD3, HOPX, PRKAR1A, TPM1, CDKN1B | |
|  | development of blood vessel | | 4.84E-02 | 2.028 | 22 |  |  |
|  | development of cardiovascular system | | 3.04E-02 | 2.028 | 28 |  |  |
| **Nutrient metabolism** | metabolism of polysaccharide | | 4.84E-02 | -0.106 | 10 | CHST1, CHST3, CHSY1, FITM2, FOXC1, GALNT5, PPARD, SMAD3, SOCS3, XYLT1 | |
| **Genetic information and nucleic acid processing** | transcription of DNA | | 4.20E-02 | -0.049 | 35 | AFF3, ARF4, ATN1, CDKN1B, DACT1, EBF3, ELK3, ETV5, FOXC1, FOXP4 | |
| **L10U** |  |  | |  |  |  |  |
| **No data above Threshold** |  |  | |  |  |  |  |
| **L10D** |  |  | |  |  |  |  |
| **Cell maintenance, proliferation differentiation and replacement** | organization of sarcomere | | 2.49E-02 |  | 5 | CAPN3, MYPN, NEB, TTN, XIRP1, PLK4 | |
|  | organization of cells | | 2.90E-02 |  | 6 |  |  |
| **Organismal, organ and tissue development** | formation of muscle cells | | 1.22E-02 | -1.964 | 11 | CAPN3, HSP90B1, NTF3, PTPN11, TTN, FHL2, HIF1A, MYH2, MYPN, NEB | |
|  | differentiation of muscle | | 2.90E-02 | -1.193 | 13 |  |  |
|  | formation of muscle | | 8.09E-03 | -0.928 | 18 |  |  |

*at maximum 10 genes are shown
