# Supplementary material for: Transient Shifts of Incubation Temperature Reveal Immediate and Long-Term Transcriptional Response in Chicken Breast Muscle Underpinning Resilience and Phenotypic Plasticity
Source: PLoS One. 2016 Sep 9;11(9):e0162485. doi: 10.1371/journal.pone.0162485 (PMC5017601; doi:10.1371/journal.pone.0162485)
Supplement: S4 Table — (DOCX) [file pone.0162485.s008.docx]

**S4** **Table. Assignment of DEGs to biological functions (major categories and Ingenuity-biofunctions) (*p*≤0.05) obtained at embryonic stage for late treatment; H13UΔC, H13DΔC, L13UΔC and L13DΔC.**

| **Major categories** | **Ingenuity-biofunction** | | **BH p-value** | **Z-score** | **# of DEGs** | **DEGs assigned to biofunction*** | |
| --- | --- | --- | --- | --- | --- | --- | --- |
| **H13U** |  |  | |  |  |  |  |
| **No data above threshold** |  |  | |  |  |  |  |
| **H13D** |  |  | |  |  |  |  |
| **Cell maintenance, proliferation differentiation and replacement** | development of cytoplasm | | 4.26E-02 | -1.264 | 6 | ANG, ARHGAP18, CNP, DYNLL1, MAPRE1, mir-17 | |
|  | formation of cytoskeleton | | 4.26E-02 | -0.832 | 5 |  |  |
|  | formation of filaments | | 4.26E-02 | -0.832 | 5 |  |  |
| **Organismal, organ and tissue development** | development of follicular epidermis | | 4.26E-02 |  | 1 | mir-17, TNFSF10, CNTNAP2, KCNA1, DYNLL1 | |
|  | end diastolic pressure of right ventricle | | 4.26E-02 |  | 1 |  |  |
|  | brain wave | | 4.26E-02 |  | 2 |  |  |
|  | quantity of fibroblast cell lines | | 4.33E-02 |  | 2 |  |  |
|  | behavioral flexibility | | 4.33E-02 |  | 1 |  |  |
| **Nutrient metabolism** | absorption of cholesterol | | 4.33E-02 |  | 2 | ACAT2, TNFSF10, CNP, NAA40, PCNA | |
|  | uptake of lipid | | 4.87E-02 |  | 3 |  |  |
|  | incorporation of adenine | | 4.66E-02 |  | 1 |  |  |
|  | induction of cyclic GMP | | 4.87E-02 |  | 1 |  |  |
| **Genetic information and nucleic acid processing** | mismatch repair | | 4.26E-02 |  | 2 | PCNA, RPA2, ANG | |
|  | mutagenesis of gene | | 4.26E-02 |  | 1 |  |  |
|  | binding of plasmid DNA | | 4.33E-02 |  | 1 |  |  |
|  | replication of DNA lesion | | 4.66E-02 |  | 1 |  |  |
|  | cleavage of tRNA | | 4.87E-02 |  | 1 |  |  |
| **Cell signaling and interaction** | autoimmune response of T lymphocytes | | 4.26E-02 |  | 1 | ANG, CNP, DYNLL1, mir-146, NMRAL1, TNFSF10 | |
|  | activation of Phospholipase A2 | | 4.33E-02 |  | 1 |  |  |
|  | synthesis of nitric oxide | | 4.66E-02 |  | 4 |  |  |
| **Small molecule biochemistry** | catabolism of cyanate | | 4.33E-02 |  | 1 | MPST | |
| **Response to stimuli** | activation of myeloid cells | | 4.66E-02 |  | 4 | CX3CR1, mir-146, mir-17, TNFSF10 | |
| **L13U** |  |  | |  |  |  |  |
| **Cell maintenance, proliferation differentiation and replacement** | arrest in cell cycle progression | | 4.45E-03 |  | 24 | AMER1, ARID1A, ARPC5, C1QBP, CCNE2, CCNG1, CDC25A, DDX17, FASN, IP6K1 | |
| **Organismal, organ and tissue development** | organismal death | | 6.64E-04 | -10.829 | 144 | CCNB2, CCNE2, EPHB3, NFE2L1, POGZ, RAD51B, SLC20A1, TP53BP2, UNG, WLS | |
|  | development of cardiovascular system | | 3.08E-02 | 2.792 | 58 | ATP2A2, B4GALT1, BACE1, CISD2, CLIC4, DHCR7, FBLN1, GNA11, IRS1, ITPA | |
|  | size of body | | 2.79E-02 | 7.091 | 55 |  |  |
| **Nutrient metabolism** | metabolism of carbohydrate | | 3.52E-02 | 3.019 | 42 | AKR1A1, B4GALT1, CTBP2, GNRH1, INPP5E, NCS1, PIP5K1C, SLC27A1, UGDH, VHL | |
|  | synthesis of carbohydrate | | 4.91E-02 | 3.391 | 31 |  | |
| **Genetic information and nucleic acid processing** | N-glycosylation of protein | | 4.50E-02 |  | 5 | ACLY, ATP5L, B4GALT1, DAD1, DDX5, GTF2H3, NUDT15, PIF1, RBBP4, STT3A | |
|  | catabolism of nucleoside triphosphate | | 4.50E-02 |  | 14 |  |  |
| **molecular transport** | internalization of protein | | 2.39E-02 | 1.264 | 11 | AP2B1, CAP1, DNM1, GLI3, IPO9, JUP, NUP214, NUTF2, PDIA3, PEX10 | |
| **L13D** |  |  | |  |  |  |  |
| **Cell maintenance, proliferation differentiation and replacement** | proliferation of cells | | 3.21E-02 | -1.768 | 10 | NOV, RUNX2, ANG, CD226, FGF6, mir-103, mir-27, PTN, TNMD, TTPA | |
|  | invasion of cells | | 2.06E-02 | -1.279 | 5 |  |  |
|  | differentiation of cells | | 4.83E-02 | -0.802 | 6 |  |  |
|  | proliferation of muscle cells | | 2.06E-02 | 0 | 4 | ANG, FGF6, mir-27, NOV | |
| **Organismal, organ and tissue development** | angiogenesis | | 4.21E-02 | -0.313 | 4 | ANG, FGF6, mir-103, TNMD | |
| **Nutrient metabolism** | activation of heparin | | 2.06E-02 |  | 1 | A4GALT, ANG, mir-27, PTN, TTPA | |
|  | metabolism of vitamin E | | 2.06E-02 |  | 1 |  |  |
|  | quantity of globotetraosylceramide | | 2.35E-02 |  | 1 |  |  |
|  | quantity of globotriaosylceramide | | 2.59E-02 |  | 1 |  |  |
|  | synthesis of acylglycerol | | 2.99E-02 |  | 2 |  |  |
| **Genetic information and nucleic acid processing** | cleavage of tRNA | | 2.59E-02 |  | 1 | ANG, RUNX2 | |
|  | binding of Runx2 binding site | | 2.86E-02 |  | 1 |  |  |
|  | cleavage of RNA fragment | | 4.04E-02 |  | 1 |  |  |
|  | transcription of rRNA | | 4.67E-02 |  | 1 |  |  |
| **Cell signaling and interaction** | activation of Phospholipase A2 | | 2.06E-02 |  | 1 | ANG, CD226, FGF6, NOV, PTN | |
|  | recognition of dendritic cells | | 2.06E-02 |  | 1 |  |  |
|  | transmembrane receptor protein tyrosine phosphatase signaling pathway | | 3.28E-02 |  | 1 |  |  |
|  | activation of muscle cells | | 2.06E-02 |  | 2 |  |  |
|  | adhesion of rhabdomyosarcoma cell lines | | 2.35E-02 |  | 1 |  |  |
| **Response to stimuli** | inflammation of urinary bladder | | 3.84E-02 |  | 1 | CHRNB4 | |

*at maximum 10 genes are shown
