# Supplementary material for: Transient Shifts of Incubation Temperature Reveal Immediate and Long-Term Transcriptional Response in Chicken Breast Muscle Underpinning Resilience and Phenotypic Plasticity
Source: PLoS One. 2016 Sep 9;11(9):e0162485. doi: 10.1371/journal.pone.0162485 (PMC5017601; doi:10.1371/journal.pone.0162485)
Supplement: S5 Table — (DOCX) [file pone.0162485.s009.docx]

**S5 Table.** **Assignment of DEGs to biological functions (major categories and Ingenuity-biofunctions) (*p*≤0.05) obtained at adult stage for early treatment; H10UΔC, H10DΔC, L10UΔC and L10DΔC.**

| **Major categories** | **Ingenuity-biofunction** | | **BH p-value** | **Z-score** | **# of DEGs** | **DEGs assigned to biofunction*** | | |
| --- | --- | --- | --- | --- | --- | --- | --- | --- |
| **H10U** |  |  | |  |  | |  |  |
| **Cell maintenance, proliferation differentiation and replacement** | fragmentation of microtubules | | 0.00679 |  | 1 | TUBB, NOG, mir-451, DPAGT1, NDFIP2 | | |
|  | differentiation of subependymal cells | | 0.00679 |  | 1 |  |  |  |
|  | maturation of erythroblasts | | 0.0114 |  | 1 |  |  |  |
|  | morphology of squamous cell carcinoma cell lines | | 0.0244 |  | 1 |  |  |  |
|  | assembly of intercellular junctions | | 0.0294 |  | 2 |  |  |  |
| **Organismal, organ and tissue development** | formation of rib | | 4.09E-03 |  | 1 | NOG, HES7, TRAF4, mir-451, TBC1D4 | | |
|  | structure of trabecular bone | | 6.79E-03 |  | 2 |  |  |  |
|  | maturation of connective tissue | | 6.79E-03 |  | 2 |  |  |  |
|  | induction of muscle | | 1.14E-02 |  | 1 |  |  |  |
|  | mass of parametrial fat pad | | 2.94E-02 |  | 2 |  |  |  |
| **Nutrient metabolism** | biosynthesis of dolichol | | 0.00679 |  | 1 | DPAGT1, TBC1D4 | | |
|  | synthesis of oligosaccharide-diphosphodolichol | | 0.0184 |  | 1 |  |  |  |
|  | metabolism of UDP-N-acetylglucosamine | | 0.0218 |  | 1 |  |  |  |
|  | homeostasis of glycogen | | 0.0373 |  | 1 |  |  |  |
| **Genetic information and nucleic acid processing** | N-glycosylation of protein | | 4.95E-02 |  | 1 | DPAGT1 | | |
| **H10D** |  |  | |  |  | |  |  |
| **Cell maintenance, proliferation differentiation and replacement** | entry into S phase of epithelial cell lines | | 2.72E-02 |  | 3 | ABL1, CHRNA7, MYC | | |
| **L10U** |  | |  |  |  |  | | |
| **Cell maintenance, proliferation differentiation and replacement** | ion homeostasis of cells | | 4.01E-02 | 2 | 6 | CHRM3, EPCAM, ERBB3, FGFBP1, mir-25, ATIC, BDKRB2, CHRM2, CLDN2, FABP1 | | |
|  | cell cycle progression | | 3.21E-02 | 0.882 | 8 |  |  |  |
|  | colony formation | | 1.18E-02 | 0.152 | 6 |  |  |  |
| **Organismal, organ and tissue development** | contractility of ileal smooth muscle | | 3.98E-03 |  | 2 | NOG, BDKRB2, ALB, AVPR2, CHRM2, CHRM3, CLDN2, ERBB3, GCNT1, HOXA1 | | |
|  | quantity of neural crest cells | | 9.62E-03 |  | 2 |  |  |  |
|  | osmolality of urine | | 9.67E-03 |  | 3 |  |  |  |
|  | induction of muscle | | 2.89E-02 |  | 1 |  |  |  |
|  | development of body trunk | | 3.01E-02 |  | 9 |  |  |  |
| **Nutrient metabolism** | synthesis of polyols | | 9.62E-03 |  | 4 |  | | |
|  | transport of palmitic acid | | 1.70E-02 |  | 2 |  | | |
|  | hydrolysis of phosphatidylinositol | | 2.13E-02 |  | 3 |  | | |
|  | biosynthesis of dolichol | | 2.13E-02 |  | 1 |  | | |
|  | export of lysophosphatidic acid | | 2.13E-02 |  | 1 |  | | |
| **Genetic information and nucleic acid processing** | cleavage of core promoter | | 2.89E-02 |  | 1 | NME2, BDKRB2, ALB | | |
|  | mutation of mitochondrial DNA | | 3.61E-02 |  | 1 |  |  |  |
|  | binding of Hnf1 binding site | | 4.64E-02 |  | 1 |  |  |  |
| **Molecular transport** | export of molecule | | 2.57E-02 |  | 3 | ALB, FABP1, NME2 | | |
|  | leakage of H+ | | 4.64E-02 |  | 1 |  |  |  |
|  | export of snRNA | | 4.64E-02 |  | 1 |  |  |  |
| **Cell signaling and interaction** | muscarinic acetylcholine receptor, phospholipase C activating pathway | | 6.09E-03 |  | 2 | CHRM2, BDKRB2, AVPR2, TRIM36, CHRM3, TRIM7 | | |
|  | acetylcholine receptor signaling, muscarinic pathway | | 1.04E-02 |  | 2 |  |  |  |
|  | binding of guanosine 5'-O-(3-thiotriphosphate) | | 2.13E-02 |  | 3 |  |  |  |
|  | G-protein signaling, coupled to cAMP nucleotide second messenger | | 2.89E-02 |  | 2 |  |  |  |
|  | response of epithelial cell lines | | 3.61E-02 |  | 2 |  | | |
| **Small molecule biochemistry** | auto-oxidation of homocysteine | | 2.13E-02 |  | 1 | ALB, NADH, ALB | | |
|  | oxidation of xanthine | | 2.89E-02 |  | 1 |  |  |  |
|  | hydrolysis of olmesartan medoxomil | | 2.13E-02 |  | 1 |  |  |  |
| **Response to stimuli** | inflammation of body region | | 2.86E-02 | -0.849 | 9 | ALB, BDKRB2, CHRM2, CHRM3, FABP1, mir-221, mir-25, mir-302, TRAF4 | | |
| **L10D** |  |  | |  |  | |  |  |
| **Cell maintenance, proliferation differentiation and replacement** | arrest in cell cycle progression | | 9.25E-03 |  | 23 | CDKN1B, RB1, FGF2, LMNA, MYC, MYOD1, RBL2, AMER1, C1QBP, CUL5 | | |
|  | interphase of skin cell lines | | 4.36E-02 |  | 4 |  |  |  |
|  | cell cycle progression of muscle cell lines | | 4.62E-02 |  | 5 |  |  |  |
| **Genetic information and nucleic acid processing** | transcription | | 9.25E-03 | -2.029 | 101 | PPARGC1A, C1QBP, CDKN1B, E2F1, FGF2, ING4, KHDRBS1, MYC, MYOD1, PDGFB | | |
|  | transcription of RNA | | 1.47E-02 | -1.665 | 98 |  |  |  |
|  | transcription of DNA | | 3.91E-02 | -0.815 | 79 |  |  |  |
|  | processing of mRNA | | 9.25E-03 | -0.263 | 17 |  |  |  |
|  | DNA replication | | 1.78E-02 | -0.239 | 21 |  |  |  |

*at maximum 10 genes are shown
