# Supplementary material for: Transient Shifts of Incubation Temperature Reveal Immediate and Long-Term Transcriptional Response in Chicken Breast Muscle Underpinning Resilience and Phenotypic Plasticity
Source: PLoS One. 2016 Sep 9;11(9):e0162485. doi: 10.1371/journal.pone.0162485 (PMC5017601; doi:10.1371/journal.pone.0162485)
Supplement: S6 Table — (DOCX) [file pone.0162485.s010.docx]

**S6 Table. Assignment of DEGs to biological functions (major categories and Ingenuity-biofunctions) (*p*≤0.05) obtained at adult stage for late treatment; H13UΔC, H13DΔC, L13UΔC and L13DΔC.**

| **Major categories** | **Ingenuity-biofunction** | | **BH p-value** | | **Z-score** | | **# of DEGs** | **DEGs assigned to biofunction*** | | |
| --- | --- | --- | --- | --- | --- | --- | --- | --- | --- | --- |
| **H13U** |  |  | |  | |  | | |  |  |
| **Genetic information and nucleic acid processing** | binding of homeodomain binding site | | 5.11E-03 | |  | | 3 | HOXA10, HOXA9, PRKCQ | | |
| **H13D** |  |  | |  | |  | | |  |  |
| **Cell maintenance, proliferation differentiation and replacement** | invasion of carcinoma cell lines | | 4.68E-02 | | -1.982 | | 4 | DSE, ID1, mir-214, NRP2 | | |
| **Organismal, organ and tissue development** | size of body | | 2.91E-02 | | -3.809 | | 15 | ID1, IL15, B4GALT1, CX3CL1, FOXA2, GSTZ1, RGN, ADRA1A, ALB, NQO1 | | |
|  | development of body trunk | | 1.94E-02 | | -0.798 | | 18 |  |  |  |
|  | quantity of monocytes | | 2.91E-02 | | -0.651 | | 5 |  |  |  |
|  | quantity of phagocytes | | 4.13E-02 | | 0.36 | | 9 | B4GALT1, CHRNA7, GNMT, ID1, IL15, NQO1, TRAF3IP2, CX3CL1, FABP1 | | |
|  | quantity of neutrophils | | 2.91E-02 | | 0.431 | | 7 |  |  |  |
| **Nutrient metabolism** | quantity of carbohydrate | | 1.94E-02 | | -3.159 | | 13 | FOXA2, GNMT, PTGER4, SLC27A1, ADRA1A, B4GALT1, CX3CL1, DSE, NQO1, TRHR | | |
|  | metabolism of carbohydrate | | 1.94E-02 | | -2.613 | | 14 |  |  |  |
|  | synthesis of carbohydrate | | 2.91E-02 | | -2.608 | | 10 |  |  |  |
|  | uptake of lipid | | 3.53E-02 | | -1.476 | | 6 |  |  |  |
|  | concentration of lipid | | 2.91E-02 | | -1.276 | | 15 |  |  |  |
| **Genetic information and nucleic acid processing** | homotetramerization of protein | | 2.08E-02 | |  | | 4 | GNMT, PCBD1, PPAT, SHMT1 | | |
| **Molecular transport** | quantity of NADH | | 3.46E-02 | |  | | 2 | CX3CL1, NQO1, SLC27A1 | | |
|  | secretion of lactic acid | | 2.91E-02 | |  | | 2 |  |  |  |
| **Small molecule biochemistry** | metabolism of pyruvic acid | | 3.53E-02 | |  | | 3 | NADH, NQO1, SLC27A1 | | |
| **L13U** | | | | | | | | | | |
| **Cell maintenance, proliferation differentiation and replacement** | apoptosis of muscle cells | | 3.49E-02 | | -0.956 | | 7 | MYC, CASP1, CAV3, IGF1R, APOA1, BID, CDKN1B, S100A6, TFPI2, TNNT2 | | |
|  | cell death of muscle cells | | 9.15E-03 | | -0.664 | | 10 |  |  |  |
|  | cell death of breast cell lines | | 5.46E-03 | | -0.447 | | 5 |  |  |  |
|  | size of muscle cells | | 1.92E-02 | | -0.447 | | 5 |  |  |  |
|  | apoptosis of B lymphocytes | | 3.49E-02 | | -0.084 | | 4 |  |  |  |
|  | proliferation of muscle cells | | 1.29E-02 | | 1.123 | | 10 | CDKN1B, IGF1R, MYC, CEBPB, TGM2, NPY, RHOB, APOD, BID, CLU | | |
|  | proliferation of cells | | 3.10E-03 | | 1.836 | | 45 |  |  |  |
|  | proliferation of epithelial cells | | 1.79E-02 | | 1.841 | | 8 |  |  |  |
|  | proliferation of fibroblast cell lines | | 3.49E-02 | | 2.018 | | 9 |  |  |  |
|  | proliferation of connective tissue cells | | 1.92E-02 | | 2.035 | | 8 |  |  |  |
| **Organismal, organ and tissue development** | quantity of leukocytes | | 1.29E-02 | | -1.275 | | 17 | CDKN1B, APOA1, CEBPB, CISH, IGF1R, MR1, MYC, NFKB2, NPY, P2RX5 | | |
|  | quantity of blood cells | | 1.12E-02 | | -1.062 | | 18 |  |  |  |
|  | quantity of T lymphocytes | | 2.90E-02 | | -0.181 | | 11 |  |  |  |
|  | quantity of brown adipose tissue | | 9.15E-03 | | -0.152 | | 4 |  |  |  |
|  | proliferation of mammary cells | | 4.75E-03 | | 1.715 | | 4 | CAV3, CSRP3, DES, TNNT2, IGF1R, TGM2, APOA1, CLU, MYL3, CEBPB | | |
|  | contractility of heart ventricle | | 2.06E-02 | | 1.98 | | 4 |  |  |  |
|  | contractility of cardiac muscle | | 3.43E-02 | | 2.144 | | 5 |  |  |  |
|  | formation of muscle | | 6.51E-06 | | 2.219 | | 16 |  |  |  |
|  | contractility of heart | | 7.49E-03 | | 2.364 | | 7 |  |  |  |
| **Nutrient metabolism** | accumulation of lipid | | 2.26E-02 | | -0.406 | | 8 | CAV3, CEBPB, FABP1, SLC27A1, APOD, CASP1, MYC, BID, NPY, PSAP | | |
|  | concentration of fatty acid | | 2.59E-03 | | -0.125 | | 10 |  |  |  |
|  | concentration of triacylglycerol | | 1.38E-02 | | -0.012 | | 9 |  |  |  |
|  | binding of lipid | | 3.49E-02 | | 1.698 | | 4 | FABP1, ALB, FABP3, APOA1, PSAP, SLC27A1, CLU, MYC, CASP1, CEBPB | | |
|  | transport of palmitic acid | | 3.20E-04 | | 1.955 | | 4 |  |  |  |
|  | transport of lipid | | 7.24E-03 | | 2.389 | | 7 |  |  |  |
|  | quantity of glycogen | | 2.59E-03 | | 2.412 | | 6 |  |  |  |
|  | fatty acid metabolism | | 3.49E-02 | | 2.837 | | 11 |  |  |  |
| **Genetic information and nucleic acid processing** | quantity of protein in blood | | 3.49E-02 | | -0.108 | | 10 | APOA1, CAV3, CDKN1B, CEBPB, DIO3, LECT1, PPIA, SAT1, SSTR2, TGM2 | | |
|  | synthesis of protein | | 3.49E-02 | | 1.009 | | 9 | CAV3, CDKN1B, GPC1, MYC, NPY, PABPC1, RPS3, SAT1, VHL | | |
| **Molecular transport** | transport of molecule | | 3.49E-02 | | 2.414 | | 23 | ALB, ANXA2, APOA1, CACNA2D2, CASP1, CDKN1B, CHRND, CHRNG, CLU, FABP1 | | |
| **Cell signaling and interaction** | interaction of colon carcinoma cells | | 3.49E-02 | |  | | 1 | TSTA3 | | |
| **Small molecule biochemistry** | oxidation of palmitic acid | | 1.99E-04 | | 1.572 | | 6 | APOA1, BID, FABP1, IGF1R, SAT1, SLC27A1, FABP3 | | |
|  | oxidation of long chain fatty acid | | 1.06E-04 | | 1.8 | | 7 |  |  |  |
| **Response to stimuli** | inflammation of body region | | 9.57E-05 | | -3.733 | | 24 | APOA1, CAV3, CLU, PPIA, ALB, BID, CASP1, CHRND, CHRNG, CISH | | |
|  | inflammation of organ | | 1.77E-04 | | -3.462 | | 26 |  |  |  |
|  | inflammation of heart | | 4.57E-03 | | -1.98 | | 4 |  |  |  |
|  | inflammation of body cavity | | 4.09E-03 | | -1.83 | | 14 |  |  |  |
| **L13D** | | | | | | | | | | |
| **Organismal, organ and tissue development** | size of body | | 7.55E-03 | | -4.121 | | 21 | AKAP6, CNTNAP2, DNAJA1, EYA4, GNPAT, GPD2, GSTZ1, HOXD4, PPP1R3A, SLC25A25 | | |

*at maximum 10 genes are shown
